# Efficacy of Panax notoginseng saponins on functional outcome in obese patients with acute ischemic stroke

**DOI:** 10.1016/j.jgr.2026.100991

**Published:** 2026-02-06

**Authors:** Sijin Wang, Peipei Du, Longfei Wu, Ziwen Xu, Yixuan Li, Ying Gao, Xunming Ji, Haiqing Song, Chi Zhang

**Affiliations:** a Dongzhimen Hospital, Beijing University of Chinese Medicine, Beijing, China; b Department of Neurology, Xuanwu Hospital, Capital Medical University, Beijing, China; c Hong Kong Baptist University, Hong Kong, China; d Institute for Brain Disorders, Dongzhimen Hospital, Beijing University of Chinese Medicine, Beijing, China; e Peking Union Medical College, Beijing, China

**Keywords:** Acute ischemic stroke, Panax notoginseng saponins, Abdominal obesity, Obesity

## Abstract

**Background:**

Obesity exacerbates acute ischemic stroke (AIS) outcomes through metabolic dysfunction and chronic inflammation. Although *Panax notoginseng* saponins (PNS) have demonstrated efficacy in the treatment of AIS in the large-scale PANDA trial (N = 3,072; ChiCTR1800016363), their benefits in patients with obesity specifically, remain unclear. This study aimed to evaluate the effect of PNS on functional outcomes in patients with obesity.

**Methods:**

This analysis utilized individual patient data from the PANDA trial. Participants were stratified by body mass index (BMI), waist circumference (WC), and a combination of both metrics. The primary outcome was functional independence, defined as a modified Rankin Scale (mRS) score of 0–2 at 90 days. Adjusted odds ratios (aORs) were calculated using multivariable logistic regression.

**Results:**

Among 2,779 patients (mean age 60.7 ± 9.3 years), 58.3% were classified as overweight or obese by BMI, and 65% met criteria for abdominal obesity based on WC. PNS significantly improved rates of functional independence at 90 days across overweight (aOR = 2.05; 95% CI: 1.39–3.06), obesity (aOR = 2.18; 95% CI: 1.11–4.41), and abdominal obesity (aOR = 2.37; 95% CI: 1.73–3.28) subgroups. Consistent benefits were observed in patients with abdominal obesity irrespective of BMI category: lower BMI (aOR = 2.45; 95% CI: 1.34–4.61) and higher BMI (aOR = 2.40; 95% CI: 1.64–3.54).

**Conclusions:**

These results indicate that PNS may improve 90-day functional outcomes in patients with AIS and obesity, including those with abdominal obesity, warranting further prospective validation.

## Graphical abstract

**Figure:**
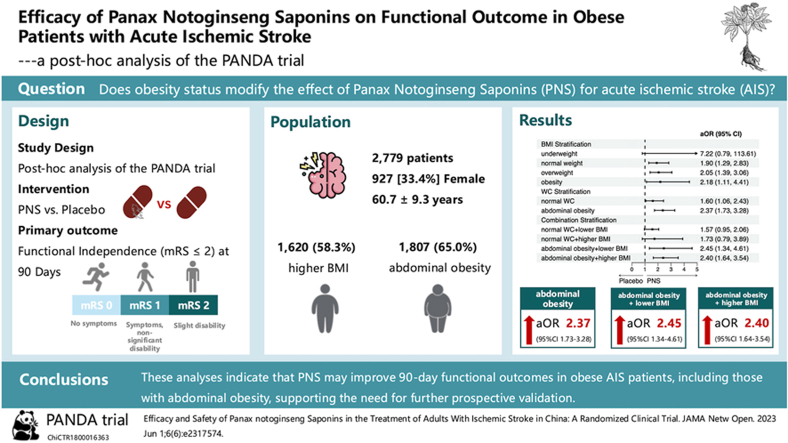

## 1 Introduction

Acute ischemic stroke (AIS) is a leading cause of mortality and disability worldwide [1]⁠. Concurrently, obesity has become a critical public health challenge, affecting more than two billion people globally [2]⁠. Evidence indicates that obesity increases the risk of stroke and adversely affects functional recovery post-stroke [[3], [4], [5]]. Particularly, abdominal obesity, characterized by excessive visceral adipose tissue, has been a well-established independent risk factor for stroke [6]. Recent studies suggest that abdominal obesity is associated with poor neurological outcomes after AIS [7]. This impaired recovery is often attributed to chronic inflammation, endothelial dysfunction, and metabolic disturbances such as insulin resistance, which are hallmarks of obesity that can exacerbate secondary brain injury and hinder neurorepair [[8], [9], [10], [11], [12]]. Given the escalating prevalence of obesity and its compounding effect on stroke-related disability, identifying strategies to improve functional recovery in this population represents an urgent clinical priority.

*Panax notoginseng* (Burk.) F.H. Chen, which is referred to as Sanqi in China, is a perennial herb belonging to the Araliaceae family. It has been used in traditional medicine for more than 400 years. *Panax notoginseng* saponins (PNS), the main bioactive constituents of this herb, are widely employed for the treatment of cardiovascular and cerebrovascular diseases. PNS includes several saponins such as ginsenosides Rg1, Rd, Rb1, Re, and notoginsenoside R1, which mediate therapeutic effects through anti-inflammatory [13], antioxidant [14,15], pro-neurogenic [16], and pro-angiogenic [17] mechanisms following cerebral ischemia. These multifaceted mechanisms have established PNS as a compound of significant interest in pharmacological research worldwide. More recently, PNS has garnered attention for its potential metabolic benefits, including improved insulin sensitivity and modulation of obesity-related pathological processes, suggesting its potential applicability in patients with obesity and AIS [[18], [19], [20]]. The PANDA trial (N = 3,072), which is the largest multicenter randomized controlled trial of PNS in patients with AIS to date, demonstrated improved functional outcomes at 90 days without increasing adverse events [21]. Although the main PANDA trial established the overall efficacy of PNS in patients with AIS, it remains unclear whether this benefit extends uniformly across the growing population of patients with obesity and stroke, who are at increased risk of suboptimal recovery. Therefore, we performed a post-hoc analysis to investigate the efficacy of PNS in patients with obesity and AIS, an inquiry not addressed in the primary trial report, with significant implications for personalized post-stroke management.

## 2 Methods

### 2.1 Study design

**Fig. 1:**
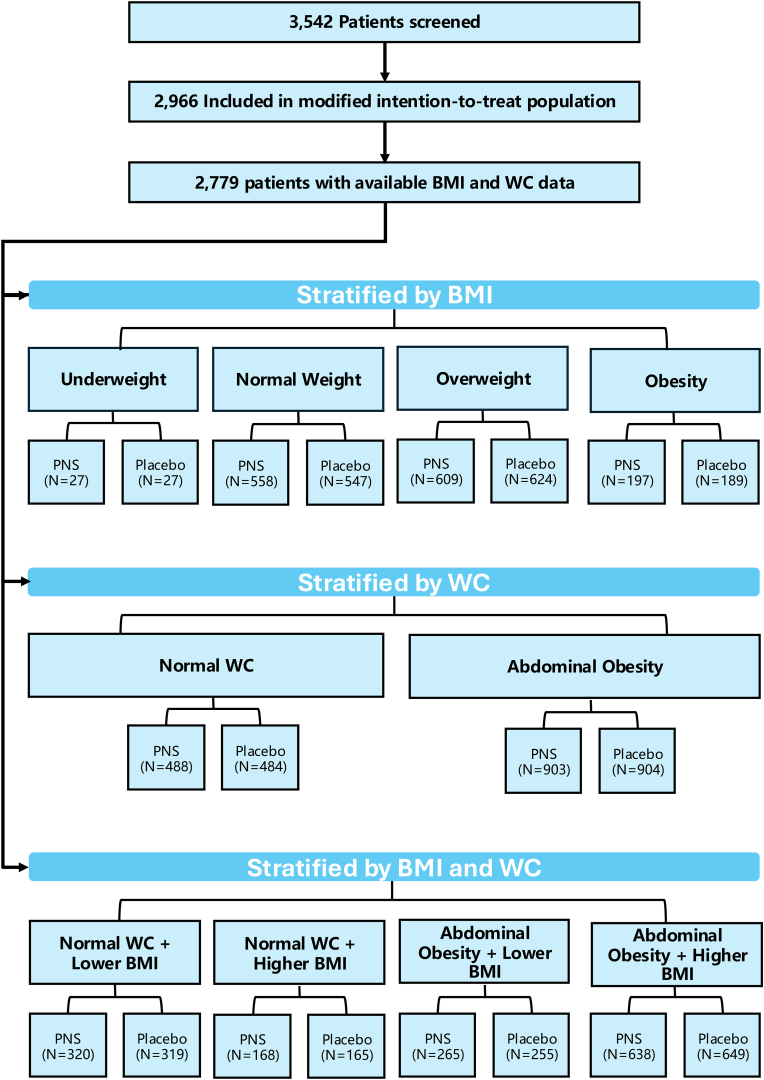
**Flow chart of patient populations. Abbreviations:** BMI, body mass index; PNS, *Panax Notoginseng* Saponins; WC, waist circumference.

We utilized individual data from the original PANDA trial, which evaluated the efficacy of PNS in ischemic stroke. The PANDA trial was a randomized, double-blind, placebo-controlled study involving 3,072 participants (Registration No.: ChiCTR1800016363; chictr.org.cn). Randomization was performed using a centralized randomization system to ensure allocation concealment. The study design and primary outcomes have been previously reported [21,22]. Further protocol details are provided in Supplementary Material 1. The study adhered to strict ethical standards, and all patients provided written informed consent before participating in any study-related procedures. Ethical approval was granted by the Ethics Committee of Xuanwu Hospital, Capital Medical University (Approval No.: LYS[2018]005). The flow chart of this study is presented in Fig. 1.

### 2.2 Population and treatment

Eligible participants were aged between 18 and 75 years, diagnosed with AIS, had a pre-stroke modified Rankin Scale (mRS) score of ≤1, and a National Institutes of Health Stroke Scale (NIHSS) score between 4 and 15 at the time of randomization. Patients were randomly assigned to either the PNS group or the placebo group.

### 2.3 Anthropometric measures

Body Mass Index (BMI) and Waist Circumference (WC) were used as the standard of overall obesity and abdominal obesity, respectively. Data on height, weight, and WC were collected after randomization. BMI was calculated as weight in kilograms divided by the square of height in meters (kg/m^2^). Criteria for BMI and WC categories were based on the Chinese Guidelines for the Clinical Management of Obesity (2024 edition). We applied Chinese BMI criteria in this study because the study population was exclusively Chinese, and Asian populations exhibit a higher percentage of body fat and increased risk of obesity-related comorbidities at a given BMI compared with other populations [23]; therefore, population-specific criteria are more accurate for risk stratification. Patients were stratified as follows: (1) According to BMI: underweight (BMI <18.5 kg/m^2^), normal weight (18.5 ≤ BMI <24 kg/m^2^), overweight (24 ≤ BMI <28 kg/m^2^), and obese (BMI ≥28 kg/m^2^); (2) According to WC: normal WC (men <85 cm, women <80 cm) and abdominal obesity (men ≥85 cm, women ≥80 cm); (3) Combined stratification: lower BMI (BMI <24 kg/m^2^) with normal WC, higher BMI (BMI ≥24 kg/m^2^) with normal WC, lower BMI (BMI <24 kg/m^2^) with abdominal obesity, and higher BMI (BMI ≥24 kg/m^2^) with abdominal obesity.

### 2.4 Outcomes

The primary efficacy outcome was the rate of functional independence, defined as mRS ≤2, at 3 months. The secondary efficacy outcomes were as follows: (1) the rate of functional independence at 12 months; and (2) the proportion of no or minimal disability, defined as mRS≤1, at 3 and 12 months.

### 2.5 Statistical analysis

All randomized participants who received at least one dose of the study drug (PNS or placebo) and had at least one assessment of the primary efficacy outcome, were included in the intent-to-treat (ITT) population. All analyses were performed on this population.

Baseline characteristics were analyzed using χ^2^ tests for categorical data and one-way analysis of variance for continuous variables, and presented as frequencies (percentages) and mean (Standard Deviation), respectively. After adjustment for sex, age, diastolic blood pressure, history of ischemic stroke, vasospastic angina, hyperlipidemia, hypertension, diabetes, and NIHSS score at randomization, multivariable logistic regression was used to assess the associations between BMI/WC categories (individually and combined) and functional outcomes, reporting adjusted odds ratios (ORs) with 95% confidence intervals (CIs). Data analysis was performed using the R software (v4.3.2). Statistical significance for all two-tailed tests was established at a P-value of <0.05.

## 3 Results

### 3.1 Baseline characteristics

Among the 2,779 patients with available BMI and WC data (927 [33.4%] women; mean age, 60.7 ± 9.3 years), 1,557 (56%) presented with functional independence (an mRS score of 0–2) at randomization. Obesity was highly prevalent: 1,620 patients (58.3%) were overweight or obese according to BMI criteria, and 1,807 (65%) met the criteria for abdominal obesity based on WC. Notably, among the 1,159 patients with a normal BMI, 520 (44.9%) had abdominal obesity. Only 536 patients (19.3%) had both, a normal BMI and a normal WC. Detailed baseline characteristics stratified by BMI, WC, and their combination are provided in Tables S1, S2, and S3.

### 3.2 Efficacy of PNS in acute ischemic stroke with comorbid obesity

**Fig. 2:**
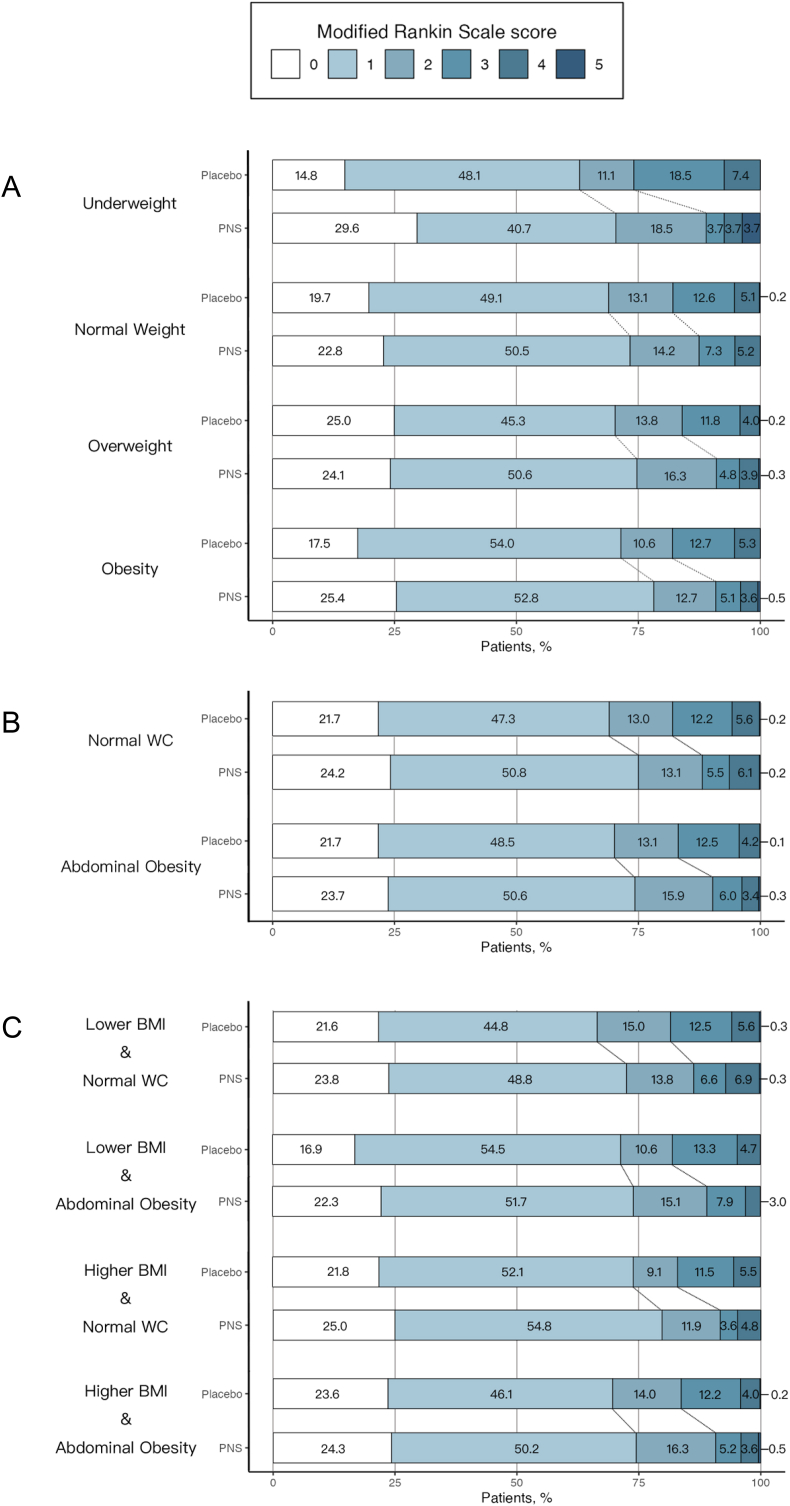
**Distribution of modified Rankin Scale (mRS) scores at 3 months. A: Body mass index (BMI) categories. B: Waist circumference (WC) categories. C: Combined BMI and WC categories.** The bars represent the percentage of patients in each mRS category (0-2: functional independence) for the *Panax Notoginseng* Saponins and placebo groups within each stratification.

The detailed primary outcomes are shown in Table 1 and Fig. 2, while the secondary outcomes are provided in Table 2.

**Table 1:** Functional Independence at 3 months.

		PNS	placebo	OR	P value for interaction	aOR	P value for interaction
		n (%)	n (%)				
**BMI Stratification**							
underweight		24 (88.9)	20 (74.1)	2.80(0.68-14.33)	0.607	7.22(0.79-113.61)	0.826
normal weight		488 (87.5)	449 (82.1)	1.52(1.09-2.13) ∗		1.90(1.29-2.83) ∗	
overweight		554 (91.0)	525 (84.0)	1.92(1.36-2.74) ∗∗∗		2.05(1.39-3.06) ∗∗∗	
obesity		179 (90.9)	155 (82.0)	2.18(1.2-4.09) ∗		2.18(1.11-4.41) ∗	
**WC Stratification**							
normal WC		430 (88.1)	397 (82.0)	1.62(1.14-2.34) ∗	0.543	1.60(1.06-2.43) ∗	0.126
abdominal obesity		815 (90.3)	752 (83.2)	1.87(1.42-2.49) ∗∗∗		2.37(1.73-3.28) ∗∗∗	
**Combination Stratification**							
normal WC	lower BMI	276 (86.2)	260 (81.5)	1.42(0.93-2.19)	0.265	1.57(0.95-2.60)	0.775
	higher BMI	154 (91.7)	137 (83.0)	2.25(1.16-4.56) ∗		1.73(0.79-3.89)	
abdominal obesity	lower BMI	236 (89.1)	209 (82.0)	1.79(1.09-2.98) ∗	0.827	2.45(1.34-4.61) ∗	0.973
	higher BMI	579 (90.8)	543 (83.7)	1.92(1.37-2.70) ∗∗∗		2.40(1.64-3.54) ∗∗∗	
lower BMI	normal WC	276 (86.2)	260 (81.5)	1.42(0.93-2.19)	0.493	1.57(0.95-2.60)	0.379
	abdominal obesity	236 (89.1)	209 (82.0)	1.79(1.09-2.98) ∗		2.45(1.34-4.61) ∗	
higher BMI	normal WC	154 (91.7)	137 (83.0)	2.25(1.16-4.56) ∗	0.680	1.73(0.79-3.89)	0.405
	abdominal obesity	579 (90.8)	543 (83.7)	1.92(1.37-2.70) ∗∗∗		2.40(1.64-3.54) ∗∗∗	

**Abbreviations:** aOR, adjusted odds ratio; BMI, body mass index; OR, odds ratio; PNS, *Panax Notoginseng* Saponins; WC, waist circumference.

aOR was adjusted for sex, age, diastolic blood pressure (DBP), history of ischaemic stroke, vasospastic angina (VSA), hyperlipidemia, hypertension, diabetes, and National Institutes of Health Stroke Scale (NIHSS) at randomization.

∗P value < 0.05; ∗∗P value < 0.01; ∗∗∗P value < 0.001.

**Table 2:** Secondary efficacy outcomes.

		PNS	placebo	OR	P for interaction	aOR	P for interaction
		n (%)	n (%)				
**mRS score** ≤ **2 at 12 months**							
**BMI Stratification**							

underweight		26 (96.3)	21 (77.8)	7.43(1.15-146.17) ∗	0.300	7.72(0.59-272.23)	0.561
normal weight		509 (91.2)	493 (90.1)	1.14(0.76-1.71)		1.22(0.78-1.90)	
overweight		573 (94.1)	580 (92.8)	1.23(0.79-1.95)		1.19(0.73-1.95)	
obesity		180 (91.4)	169 (89.4)	1.25(0.64-2.50)		1.07(0.50-2.29)	
**WC Stratification**							
normal WC		454 (93.0)	435 (89.9)	1.50(0.96-2.39)	0.294	1.43(0.88-2.35)	0.627
abdominal obesity		834 (92.4)	828 (91.6)	1.11(0.79-1.56)		1.21(0.84-1.75)	
**Combination Stratification**							
normal WC	lower BMI	292 (91.2)	281 (88.1)	1.41(0.85-2.38)	0.588	1.52(0.86-2.70)	0.929
	higher BMI	162 (96.4)	154 (93.3)	1.93(0.72-5.72)		1.49(0.46-5.11)	
abdominal obesity	lower BMI	243 (91.7)	233 (91.4)	1.04(0.56-1.94)	0.811	1.06(0.54-2.10)	0.808
	higher BMI	591 (92.6)	595 (91.7)	1.14(0.76-1.72)		1.23(0.80-1.91)	
lower BMI	normal WC	292 (91.2)	281 (88.1)	1.41(0.85-2.38)	0.462	1.52(0.86-2.70)	0.495
	abdominal obesity	243 (91.7)	233 (91.4)	1.04(0.56-1.94)		1.06(0.54-2.10)	
higher BMI	normal WC	162 (96.4)	154 (93.3)	1.93(0.72-5.72)	0.342	1.49(0.46-5.11)	0.856
	abdominal obesity	591 (92.6)	595 (91.7)	1.14(0.76-1.72)		1.23(0.80-1.91)	
**mRS score of 0 or 1 at 3 months**							
**BMI Stratification**							

underweight		19 (70.4)	17 (63.0)	1.40(0.45-4.46)	0.953	2.51(0.42-17.87)	0.832
normal weight		409 (73.3)	377 (68.9)	1.24(0.95-1.61)		1.37(1.02-1.86) ∗	
overweight		455 (74.7)	439 (70.2)	1.25(0.97-1.61)		1.26(0.94-1.67)	
obesity		154 (78.2)	135 (71.4)	1.43(0.90-2.28)		1.45(0.85-2.49)	
**WC Stratification**							
normal WC		366 (75.0)	334 (69.0)	1.35(1.02-1.79) ∗	0.614	1.29(0.93-1.79)	0.813
abdominal obesity		671 (74.3)	634 (70.1)	1.23(1.00-1.51) ∗		1.34(1.06-1.70) ∗	
**Combination Stratification**							
normal WC	lower BMI	232 (72.5)	212 (66.5)	1.33(0.95-1.87)	0.891	1.42(0.96-2.11)	0.369
	higher BMI	134 (79.8)	122 (73.9)	1.39(0.83-2.33)		1.05(0.57-1.94)	
abdominal obesity	lower BMI	196 (74.0)	182 (71.4)	1.14(0.77-1.68)	0.641	1.23(0.78-1.95)	0.643
	higher BMI	475 (74.5)	452 (69.6)	1.27(1.00-1.62) ∗		1.39(1.06-1.84) ∗	
lower BMI	normal WC	232 (72.5)	212 (66.5)	1.33(0.95-1.87)	0.553	1.42(0.96-2.11)	0.619
	abdominal obesity	196 (74.0)	182 (71.4)	1.14(0.77-1.68)		1.23(0.78-1.95)	
higher BMI	normal WC	134 (79.8)	122 (73.9)	1.39(0.83-2.33)	0.757	1.05(0.57-1.94)	0.370
	abdominal obesity	475 (74.5)	452 (69.6)	1.27(1.00-1.62) ∗		1.39(1.06-1.84) ∗	
**mRS score of 0 or 1 at 12 months**							
**BMI Stratification**							

underweight		22 (81.5)	20 (74.1)	1.54(0.42-5.95)	0.862	1.58(0.24-12.1)	0.887
normal weight		470 (84.2)	440 (80.4)	1.30(0.95-1.77)		1.40(1.00-1.97)	
overweight		527 (86.5)	512 (81.9)	1.42(1.04-1.94) ∗		1.45(1.04-2.05) ∗	
obesity		166 (84.3)	157 (83.1)	1.09(0.64-1.88)		0.95(0.51-1.78)	
**WC Stratification**							
normal WC		414 (84.8)	388 (80.2)	1.38(0.99-1.94)	0.726	1.32(0.91-1.91)	0.768
abdominal obesity		771 (85.4)	741 (82.0)	1.28(1.00-1.65)		1.40(1.07-1.84) ∗	
**Combination Stratification**							
normal WC	lower BMI	267 (83.4)	252 (79.0)	1.34(0.90-2.00)	0.77	1.39(0.89-2.17)	0.764
	higher BMI	147 (87.5)	136 (82.4)	1.49(0.82-2.77)		1.25(0.61-2.54)	
abdominal obesity	lower BMI	225 (84.9)	208 (81.6)	1.27(0.80-2.02)	0.954	1.31(0.78-2.18)	0.970
	higher BMI	546 (85.6)	533 (82.1)	1.29(0.96-1.74)		1.44(1.04-2.00) ∗	
lower BMI	normal WC	267 (83.4)	252 (79.0)	1.34(0.90-2.00)	0.866	1.39(0.89-2.17)	0.923
	abdominal obesity	225 (84.9)	208 (81.6)	1.27(0.80-2.02)		1.31(0.78-2.18)	
higher BMI	normal WC	147 (87.5)	136 (82.4)	1.49(0.82-2.77)	0.675	1.25(0.61-2.54)	0.531
	abdominal obesity	546 (85.6)	533 (82.1)	1.29(0.96-1.74)		1.44(1.04-2.00) ∗	

**Abbreviations:** aOR, adjusted odds ratio; BMI, body mass index; OR, odds ratio; PNS, Panax Notoginseng Saponins; WC, waist circumference.

aOR was adjusted for sex, age, diastolic blood pressure (DBP), history of ischaemic stroke, vasospastic angina (VSA), hyperlipidemia, hypertension, diabetes, and National Institutes of Health Stroke Scale (NIHSS) at randomization.

∗P value < 0.05.

#### 3.2.1 Efficacy outcomes stratified by BMI

PNS was associated with a higher proportion of functional independence, in patients with a higher BMI. Among overweight patients, the rate of functional independence was 91.0% in the PNS group compared to 84.0% in the placebo group (adjusted odds ratio [aOR] = 2.05 [95% CI, 1.39–3.06]). A similar benefit was observed in patients with obesity (90.9% vs. 82.0%; aOR = 2.18 [95% CI, 1.11–4.41]). Additionally, in the overweight group, the PNS group had a significantly higher proportion of patients with an absolute increase of 4.6% in the rate of no or minimal disability at 12 months, (86.5% vs. 81.9%; aOR = 1.45 [95% CI, 1.04–2.05]).

#### 3.2.2 Efficacy outcomes stratified by WC

Additionally, PNS was associated with significant improvements in patients with abdominal obesity. The functional independence rate was 90.3% in the PNS group versus 83.2% in the placebo group (aOR = 2.37 [95% CI, 1.73-3.28]). Furthermore, PNS treatment increased the proportion of patients with no or minimal disability at 3 months (74.3% vs. 70.1%; aOR = 1.34 [95% CI, 1.06-1.70]) and demonstrated an absolute increase of 3.4% at 12 months (85.4% vs. 82.0%; aOR = 1.40 [95% CI, 1.07–1.84]) in this group.

#### 3.2.3 Efficacy outcomes stratified by BMI and WC

The effect of PNS in improving 3-month functional independence were significant in the abdominal obesity subgroups, regardless of the BMI status (lower BMI: aOR = 2.45 [95% CI, 1.34–4.61]; higher BMI: aOR = 2.40 [95% CI, 1.64–3.54]). Conversely, no significant benefit was observed in patients without abdominal obesity across both BMI strata (lower BMI: aOR = 1.57 [95% CI, 0.95–2.60]; higher BMI: aOR = 1.73 [95% CI, 0.79–3.89]).

## 4 Discussion

The analysis in the present study yielded two principal findings: first, PNS significantly improved the rate of functional independence at 3 months in patients with overall obesity and those with abdominal obesity; second, the treatment effect was numerically higher in the abdominal obesity subgroup, suggesting a potential trend that warrants further investigation. These findings are further supported by the secondary outcome analysis focusing on longer-term recovery. PNS was associated with a higher proportion of individuals achieving no or minimal disability at 12 months, with an increase of 4.6% in the overweight subgroup and 3.4% in the abdominal obesity subgroup. These consistent benefits on long-term functional outcomes underscore the durable therapeutic effect of PNS in this clinically challenging population.

Abdominal obesity is increasingly recognized as an independent factor that compromises therapeutic efficacy across multiple treatment modalities, posing considerable challenges to optimal stroke recovery [6,24,25]. Therefore, patients in the present study were further stratified using WC to capture abdominal obesity more accurately. Notably, PNS remained effective in this analysis, demonstrating an absolute improvement of 7.1% in the rate of functional independence among patients with abdominal obesity. These concordant findings across different anthropometric measures strengthen the evidence for PNS efficacy in this high-risk population, that often demonstrates suboptimal responses to conventional therapies.

When viewed in the context of existing research, our findings hold significant importance. Previous studies have consistently demonstrated that patients with obesity experiencing AIS respond inadequately to conventional treatments. A consecutive cohort study of 304 patients receiving intravenous thrombolysis therapy showed that obesity led to a lower rate of favorable outcomes following therapy by 17.2% [26]. Furthermore, a study using abdominal computed tomography to accurately quantify visceral adipose tissue (VAT) revealed that among patients with AIS receiving guideline-recommended treatment, those in the highest VAT group had a 14.5% lower probability of achieving a favorable outcome compared with the lowest VAT group [25]. Another study focusing on abdominal fat distribution further confirmed that abdominal obesity reduces the efficacy of endovascular therapy. This condition was associated with a decrease in favorable functional outcomes by 8.5% at 90 days and 12.2% at one year [24]. In contrast to these reports of attenuated treatment responses in populations with obesity, our exploratory analysis found that PNS was associated with a significant benefit in the abdominal obesity subgroup. This marked divergence from the established pattern suggests that PNS may offer a novel therapeutic strategy.

The therapeutic benefit of PNS in patients with obesity and AIS is attributed to their multi-target pharmacological actions that counter obesity-aggravated pathophysiological processes. Notably, PNS contains ginsenosides Rg1, Rd, Rb1, Re, and notoginsenoside R1 which collectively exhibit anti-inflammatory, antioxidant, anti-apoptotic, and blood–brain barrier restorative effects [16,27,28]. Importantly, these compounds mitigate the central mechanisms of stroke injury exacerbated by obesity, such as oxidative stress and inflammation. Specifically, they reduce ROS accumulation, upregulate hypoxia-inducible factor-1α [19,29,30], and modulating cytokine expression through suppression of IL-1β and TNF-α alongside elevation of IL-10 [13]. Furthermore, PNS promotes vascular repair by enhancing angiogenesis and micro-perfusion via AMPK- and eNOS) dependent pathways [31]. Particularly, ginsenoside Rb1 demonstrates potent antioxidant activity through binding to estrogen receptor-β, thereby activating protective signaling that restores eNOS and superoxide dismutase expression [15]. Moreover, abdominal obesity is closely linked to insulin resistance, a condition that exacerbates neuroinflammation, endothelial dysfunction, and impaired recovery after stroke [9,32]. The multi-target pharmacological profile of PNS is further complemented by its potential to confer metabolic benefits [33]. These benefits, primarily mediated through the modulation of neuroprotective, anti-oxidant, anti-inflammatory, micro-circulatory, and insulin resistance pathways, are consistent with predictions from previous network pharmacology analysis by the authors (Supplementary Material 2). Additionally, preliminary reports suggest that PNS improves insulin sensitivity and regulates metabolic homeostasis [[16], [17], [18]]; however, clinical validation in stroke patients remains necessary to substantiate these mechanistic insights. In summary, the synergistic modulation of neuroinflammatory, oxidative, metabolic, and vascular repair pathways emphasize the efficacy of PNS in obese stroke patients. This multi-mechanistic action highlights the need for further investigation of PNS as a potential therapy for the population that often responds poorly to conventional treatments.

The strengths of the present study include the use of data from the PANDA trial, the largest randomized trial of PNS in AIS, which ensures high-quality and standardized data collection. To the best of our knowledge, this is the largest study to provide evidence supporting the efficacy of PNS in patients with obesity and AIS. The combined use of BMI and WC enhances the robustness of our conclusions against bias from any single anthropometric measurement.

Several limitations of the present study should be considered. First, this was a post-hoc analysis, and the investigation of obesity subgroups was not pre-specified in the original PANDA trial protocol. Therefore, the findings should be interpreted as exploratory and hypothesis-generating rather than confirmatory of a causal relationship. Second, the study relied on data from Chinese and lacked individuals with severe obesity (BMI >35 kg/m^2^), which may have affected the generalizability of these results. Third, the study lacked serial data on metabolic parameters such as BMI, WC, and lipid profiles at the 90-day follow-up. Therefore, we could not directly investigate whether the observed clinical benefits were mediated through the modulation of adiposity-related metabolic pathways. This important mechanistic question should be addressed in future prospective studies. Similarly, although waist-to-hip ratio (WHR) is a valuable surrogate marker for visceral adiposity, hip circumference was not collected in the original PANDA trial protocol. Consequently, we were unable to calculate WHR or investigate its specific association with functional outcomes in this study. Moreover, the BMI categorization in this study was based on criteria specific to the Chinese population, which may limit direct comparability with studies using international BMI standards. Finally, formal tests for interaction between treatment group and obesity status did not yield statistically significant results in either the BMI or abdominal obesity subgroups, suggesting that the beneficial effect of PNS was generally consistent across these patient categories. Nonetheless, the adjusted odds ratios indicated a trend toward a more pronounced treatment effect in patients with abdominal obesity. The absence of a significant interaction may be attributed to reduced statistical power resulting from subgroup stratification. Therefore, our observation of a potentially enhanced benefit in this population should be interpreted with caution and warrants further validation in future, adequately powered prospective studies.

## 5 Conclusion

PNS is associated with improved functional outcomes at 3 months in patients with obesity and AIS, with a potentially enhanced benefit observed in those with abdominal obesity. These exploratory findings suggest that investigating PNS as a potential adjunct strategy for this population is warranted, and requires further validation in prospective studies.

## Ethics statement

This study was registered in the Chinese Clinical Trial Registry (ChiCTR1800016363, chictr.org.cn), and all procedures involving human participants were approved by the ethics committee of Xuanwu hospital of Capital Medical University (Approval Number: LYS [2018]005) in accordance with the Helsinki declaration. A written informed consent was obtained by all participants before enrolment in the study. All the authors have agreed on this publication.

## Consent for publication statement

We confirm that the details of any images, recordings, etc can be published, and that the person(s) providing consent have been shown the article contents to be published.

## Data availability statement

Data are available from the corresponding author upon reasonable request.

## Authors’ contribution statement

Sijin Wang: Conceptualization, Formal analysis, Methodology, Validation, Visualization, Writing – review & editing, Writing – original draft; Peipei Du: Data curation, Methodology, Software, Validation, Visualization, Writing – review & editing; Longfei Wu: Methodology, Validation, Visualization, Writing – review & editing; Ziwen Xu: Formal analysis, Validation, Writing – review & editing; Yixuan Li: Methodology, Validation, Writing – review & editing; Ying Gao: Funding acquisition, Investigation, Project administration, Resources, Supervision; Xunming Ji: Investigation, Project administration, Resources, Supervision; Haiqing Song: Investigation, Project administration, Writing – review & editing, Resources, Supervision; Chi Zhang: Conceptualization, Formal analysis, Funding acquisition, Methodology, Writing – review & editing, Resources, Project administration.

## Declaration of generative AI and AI-assisted technologies in the writing process

During the preparation of this work the authors used ChatGPT 4o and Deepseek to improve language. After using this tool, the authors reviewed and edited the content as needed and take full responsibility for the content of the publication.

## Funding

We would like to thank 10.13039/501100012166National Key Research and Development Program of China (2022YFC3501104) for its supported.
