# Supplementary material for: Efficacy of Panax notoginseng saponins on functional outcome in obese patients with acute ischemic stroke
Source: J Ginseng Res. 2026 Feb 6;50(3):100991. doi: 10.1016/j.jgr.2026.100991 (PMC13149892; doi:10.1016/j.jgr.2026.100991)
Supplement: Multimedia component 1 [file mmc1.pdf]

Supplementary Material

This appendix was provided by the authors to provide reviewers and readers with more information.

Efficacy and Safety of Panax Notoginseng Saponins in the Treatment of Adults with Ischemic Stroke in China: A Randomized Placebo-Controlled Clinical Trial

Study Protocol

Study Period: From November 2017 to December 2021

Version Number: 1.2

Version Date: March 8th , 2018

Table of Contents for Study Protocol

Summary of Protocol 3
Abbreviations or Terminology 6
Main Text 7
1. Background 7
2. Hypothesis 7
3. Patients 7
3.1 Sample Size 7
3.2 Diagnostic Criteria 7
3.3 Inclusion Criteria 8
3.4 Exclusion Criteria 8
4. Outcomes 9
4.1 Primary Efficacy Outcome 9
4.2 Secondary Efficacy Outcomes 9
4.3 Primary Safety Outcome 9
4.4 Secondary Safety Outcomes 9
5. Study Design 9
5.1 Study Type 10
5.2 Randomization 10
6. Study Plan and Procedures 10
6.1 Visit Schedule 10
6.2 Protocol Compliance 12
7. Treatment 13
7.1 Identification of IMPs 13
7.2 IMP Records 14
7.3 Storage of IMPs 14
7.4 Concomitant Therapy 14
7.5 Treatment Compliance and Reliability 15
7.6 Discontinuation of IMPs 15
7.7 Withdrawal from the Study 16
8. Study Management 17
8.1 Enrollment and Randomization of Patients 17
8.2 Randomization Procedures 17
8.3 Procedures of Mistakenly Included or Randomized Patients 17
8.4 Blinding and Unblinding Procedures 17
8.5 Blind Review 18
9. Quality Control (QC) 18
9.1 Storage and Auditing of Source Data/Documents 18
9.2 Pre-study Task 19
9.3 Training of Study Center Personnel 19

9.4 Study Monitoring........19
9.5 Source Data........19
9.6 Study Progress........19
10. AEs........19
10.1 Definition of AEs........19
10.2 Observation and Records of AEs........20
10.3 Severity of AEs........20
10.4 Correlation between the AE and Drugs........20
10.5 SAEs........21
10.6 Record, Management, and Report........21
10.7 Emergency Procedures........22
11. Ethical and Regulatory Requirements........22
11.1 Independent Ethics Committee Approval........22
11.2 Patient Information and Informed Consent........22
11.3 Essential Documents........22
12. Data Management........22
12.1 Data Acquisition and CRF........22
12.2 Data Management Procedures and Tasks........23
12.3 Data Quality Management........23
13. Statistical Analysis and Sample Size........24
13.1 Analysis Cohorts........24
13.2 Statistical Analysis Methods........24
13.3 Sample Size Determination........25
14. Principles for Protocol Amendment........26
15. Principles and Criteria for Complete Termination of the Trial........26
16. Confidentiality........26
17. References........27
Appendix 1. Diagnostic Criteria for Ischemic Stroke (Excerpts)........29
Appendix 2. The modified Rankin Scale........30
Appendix 3. The National Institutes of Health Stroke Scale........31
Appendix 4. EuroQoL Group 5-Dimension Questionnaire........35
Appendix 5. Barthel Index........37

Summary of Protocol

**Title**	Efficacy and Safety of Panax Notoginseng Saponins in the Treatment of Adults with Ischemic Stroke in China: A Randomized Placebo-Controlled Clinical Trial
**Coordinating centers**	Xuanwu Hospital, Capital Medical University, Dongzhimen Hospital, Beijing University of Chinese Medicine
**Indication**	Ischemic stroke
**Study objective**	To investigate the efficacy and safety of panax notoginseng saponins in patients with ischemic stroke
**Study design**	A randomized, double-blind, placebo-controlled, multi-center, clinical study
**Sample size**	3000 patients in total (1500 patients per group)
**Inclusion and Exclusion criteria**	**Inclusion criteria:**<br>1. Aged 18–75 years;<br>2. Clinical diagnosis of ischemic stroke within 14 days of symptom onset;<br>3. Pre-stroke mRS ≤ 1;<br>4. 4 ≤ NIHSS ≤ 15;<br>5. Signed written informed consent.<br>**Exclusion criteria:**<br>1. Hemorrhage or other pathologic brain disorders such as vascular malformations, tumors, abscesses, encephalitis, or other common non-ischemic brain diseases (e.g., multiple sclerosis) confirmed by head computed tomography/magnetic resonance imaging;<br>2. Had contraindications to panax notoginseng saponins:<br>1) Known allergy history;<br>2) Known allergy to agents including aspirin, salicylates, or any other ingredients of medicine included in the study;<br>3) With a medical history of asthma caused by salicylates or substances containing salicylic acid, or non-steroidal anti-inflammatory drugs;<br>4) Active peptic ulcers;<br>5) Severe hepatic insufficiency or renal insufficiency;<br>(alanine aminotransferase > 2 × upper limit of normal, or aspartate aminotransferase > 2 × upper limit of normal; creatinine > 1.5 × upper limit of normal);<br>6) Severe cardiac failure;<br>7) With a prior medical history of coagulopathy or systemic hemorrhages;<br>8) With a prior medical history of thrombocytopenia or neutropenia;<br>9) With a prior medical history of drug-induced blood system diseases or abnormal hepatic function;<br>10) Leukopenia (< 2×10^9/L) or thrombocytopenia (< 100×10^9/L);<br>11) Female in the gestation or lactation period.

	3. With indications for anticoagulation (suspected cardioembolism, such as atrial fibrillation, prosthetic heart valve, and suspected endocarditis);<br>4. With a medical history of intracranial hemorrhage or cerebral amyloid angiopathy;<br>5. With a medical history of aneurysm (including intracranial aneurysm and peripheral aneurysm);<br>6. Confirmed or suspected acute coronary syndrome;<br>7. High-risk bradyarrhythmia such as sick sinus syndrome with second- or third-degree atrioventricular conduction block, and bradycardia-related syncope induced by not installing a cardiac pacemaker;<br>8. With a history of gastrointestinal hemorrhage within 3 months prior to randomization, or undergoing a major surgery within 30 days prior to randomization;<br>9. Expected to receive endovascular treatment or operative treatment in the next 3 months;<br>10. Required to terminate the trial drug due to other scheduled surgery or interventional treatments;<br>11. Patients had severe non-cardiovascular or cerebrovascular diseases with a life expectancy of less than 3 months;<br>12. Women of childbearing age showing negative pregnancy test but refusing to take effective contraception measures;<br>13. Currently receiving an investigational drug or being involved in a device experiment;<br>14. Participated in other drug clinical trials and received other investigated medicinal products (IMPs) within 30 days before randomization;<br>15. Unable to understand and/or comply with the study procedures and/or follow-up due to psychiatric disorders, cognitive, or mental disorders;<br>16. Patients are not eligible for this clinical trial as evaluated by the investigator.
**IMPs**	Xuesaitong soft capsules; Specification: 0.33 g/capsule; Dosage form: soft capsule<br>Manufacturer: China Resources Kunming Shenghuo Pharmaceutical Co., Ltd.<br>Xuesaitong soft capsules placebo; Specification: 0.33g/capsule; Dosage form: soft capsule<br>Manufacturer: China Resources Kunming Shenghuo Pharmaceutical Co., Ltd.
**Intervention**	Eligible patients will be randomized into an XST group and a control group at a ratio of 1:1, to receive Xuesaitong soft capsules or placebo for 3 months.<br>**XST group:**<br>Xuesaitong soft capsules 60 mg/capsule, 120 mg per time, twice daily<br>**Control group:**<br>Xuesaitong soft capsules placebo 60 mg/capsule, 120 mg per time, twice daily
**Blinding**	The IMPs and placebo will be identical in color, appearance, and packaging.
**Route of administration**	Oral

**Efficacy outcomes**	**Primary outcome:**<br>Proportion of patients with functional independence (modified Rankin scale (mRS) ≤ 2) at 3 months after randomization.<br>**Secondary outcomes:**<br>1. Proportion of patients with recurrent stroke (ischemic stroke and intracerebral hemorrhage) at 3 months and 12 months after randomization;<br>2. Proportion of patients with functional independence (mRS ≤ 2) at 12 months after randomization;<br>3. Proportion of patients with no or minimal disability (mRS ≤ 1) at 3 months and 12 months after randomization;<br>4. Change in National Institutes of Health Stroke Scale score at 3 months after randomization;<br>5. Proportion of composite cerebrovascular events (ischemic stroke, intracerebral hemorrhage, myocardial infarction, and vascular death) at 3 months and 12 months after randomization;<br>6. Difference of EuroQoL Group 5-Dimension score at 3 months and 12 months after randomization;<br>7. Change in Barthel Index score at 3 months and 12 months after randomization;<br>8. Difference in platelet counts and coagulation indicators (prothrombin time, and activated partial thromboplastin time) at 3 months after randomization.
**Safety outcomes**	**Primary safety outcome:**<br>Incidence of serious adverse events within 3 months of treatment.<br>**Secondary safety outcomes:**<br>1. Incidence of symptomatic intracranial hemorrhage within 3 months of treatment;<br>2. Incidence of all-cause mortality within 3 months of treatment;<br>3. Incidence of adverse events within 3 months of treatment.

Abbreviations or Terminology

Abbreviations or Terminology	Description
AE	Adverse Event
BI	Barthel Index
CFDA	China Food and Drug Administration (now National Medical Products Administration, NMPA)
CRA	Clinical Research Associate
CRF	Case Report Form
CT	Computed Tomography
DSMB	Data and Safety Monitoring Board
ECG	Electrocardiograph
EQ-5D	EuroQoL Group 5-Dimension
GCP	Good Clinical Practice
HR	Hazard Ratio
ICF	Informed Consent Form
IMP	Investigational Medicinal Product
MRI	Magnetic Resonance Imaging
mRS	modified Rankin Scale
NIHSS	National Institutes of Health Stroke Scale
OR	Odds Ratio
PTDV	Premature Treatment Discontinuation Visit
QC	Quality Control
SAE	Serious Adverse Event

Main Text

1. Background

Stroke is characterized by high morbidity, disability, and mortality, and has become one of the major threats to human health. The Global Burden of Disease Study showed that ischemic heart disease and stroke were the top causes of death globally between 1990 and 2010. Meanwhile, the burden of disease study in China showed that stroke was the leading cause of deaths among the Chinese in 2010, accounting for 29.4% of stroke-caused deaths worldwide. Ischemic stroke accounts for approximately 70% of all strokes. However, there is a lack of effective neuroprotective agents for ischemic stroke. Therefore, the development of a scientific and inexpensive neuroprotective agent with few adverse effects has become a research hotspot in the treatment of ischemic stroke.

Currently, the Chinese economy has entered a new era of development, with the status and role of the traditional Chinese medicine in the economic and social development gaining increasing importance. The revitalization and development of the traditional Chinese medicine are about to embrace a historic opportunity with favorable international and domestic environment while receiving popular support. In recent years, scholars have recommended the use of traditional Chinese medicine more often in the prevention and treatment of cardiovascular and cerebrovascular diseases.

Xuesaitong soft capsules (XST; trade name: LI XU WANG®) are prepared from an active ingredient - panax notoginseng saponins from the famous and precious medical herb - panax notoginseng. It was formally launched in the market in June 1999 after obtaining a new drug certificate and production approval issued by the China Food and Drug Administration (CFDA, now National Medical Products Administration, NMPA). After more than 10 years of development, production and clinical application, the safety of this product is proved to be good with low incidence of adverse events (AEs). The statistics of the National Adverse Drug Reaction Monitoring System in China showed that, from 2011 to May 2017, a total of 119 cases of AEs were reported, mostly gastrointestinal reactions, dizziness, headache, rash, anaphylactoid reactions, palpitations, and nausea. Ever since it has been marketed, XST have displayed significant protective effects in cardiovascular and cerebrovascular diseases, and have been widely used in treating ischemic stroke. The pharmacological studies proved that panax notoginseng saponin has a wide range of biological activities and can significantly reduce hematocrit and fibrinogen content in the blood to reduce the blood viscosity; Rgl, an important component of panax notoginseng saponins, can significantly inhibit platelet aggregation and stimulate a reduction of the hematocrit and fibrin content in the blood and effectively prevent thrombosis. Patients with ischemic stroke who were treated with XST in addition to conventional treatment showed significantly better recovery of neurological function and improvement of daily living ability than the control group.

This multicenter, randomized, double-blind, controlled clinical trial aims to provide more evidence on the use of medications for neuroprotection in patients with ischemic stroke to further investigate the efficacy and safety of XST.

2. Hypothesis

For patients with ischemic stroke within 14 days of onset, XST can further improve the 90-day functional outcome.

3. Patients

3.1 Sample Size

A total of 3000 eligible patients will be enrolled.

3.2 Diagnostic Criteria

The diagnostic criteria for ischemic stroke are shown in Appendix 1 in detail.

3.3 Inclusion Criteria

1. Aged 18-75 years;
2. Clinical diagnosis of ischemic stroke within 14 days of symptom onset;
3. Pre-stroke modified Rankin Scale (mRS) ≤ 1;
4. $4 \leq$ National Institutes of Health Stroke Scale (NIHSS) score ≤ 15;
5. Signed written informed consent.

3.4 Exclusion Criteria

1. Hemorrhage; or other pathologic brain disorders such as vascular malformations, tumors, abscesses, encephalitis; or other common non-ischemic brain diseases (e.g., multiple sclerosis) confirmed by head computed tomography (CT)/magnetic resonance imaging (MRI);
2. Had contraindications to panax notoginseng saponins:
    1) Known allergy history;
    2) Known allergy to agents including aspirin, salicylates, or any other ingredients of medicine included in the study;
    3) With a medical history of asthma caused by salicylates or substances containing salicylic acid, or non-steroidal anti-inflammatory drugs;
    4) Active peptic ulcers;
    5) Severe hepatic insufficiency or renal insufficiency

       (alanine aminotransferase $> 2 \times$ upper limit of normal, or aspartate aminotransferase $> 2 \times$ upper limit of normal; creatinine $> 1.5 \times$ upper limit of normal);
    6) Severe cardiac failure;
    7) With a prior medical history of coagulopathy or systemic hemorrhages;
    8) With a prior medical history of thrombocytopenia or neutropenia;
    9) With a prior medical history of drug-induced blood system diseases or abnormal hepatic function;
    10) Leukopenia ($< 2 \times 10^9$/L) or thrombocytopenia ($< 100 \times 10^9$/L);
    11) Female in the gestation or lactation period.
3. With indications for anticoagulation (suspected cardioembolism, such as atrial fibrillation, prosthetic heart valve, and suspected endocarditis);
4. With a medical history of intracranial hemorrhage or cerebral amyloid angiopathy;
5. With a medical history of aneurysm (including intracranial aneurysm and peripheral aneurysm);
6. Confirmed or suspected acute coronary syndrome;
7. High-risk bradyarrhythmia like sick sinus syndrome with second- or third-degree atrioventricular conduction block, bradycardia-related syncope induced by not installing a cardiac pacemaker;
8. With a history of gastrointestinal hemorrhage within 3 months prior to randomization, or undergoing a major surgery within 30 days prior to randomization;
9. Expected to receive endovascular treatment or operative treatment in the next 3 months;

10. Required to terminate the trial drug due to other scheduled surgery or interventional treatments;
11. Patients had severe non-cardiovascular or cerebrovascular diseases with a life expectancy of less than 3 months;
12. Women of childbearing age showing negative pregnancy test but refusing to take effective contraceptive measures;
13. Currently receiving an investigational drug or being involved in a device experiment;
14. Participated in other drug clinical trials and received other Investigational Medicinal Products (IMPs) within 30 days before randomization;
15. Unable to understand and/or comply with the study procedures and/or follow-up due to psychiatric disorders, cognitive, or mental disorders;
16. Patients are not eligible for this clinical trial as evaluated by the investigator.

4. Outcomes

4.1 Primary Efficacy Outcome

Proportion of patients with functional independence (mRS $\leq$ 2) at 3 months after randomization.

4.2 Secondary Efficacy Outcomes

1. Proportion of recurrent stroke (ischemic stroke and intracerebral hemorrhage) at 3 months and 12 months after randomization;
2. Proportion of patients with functional independence (mRS $\leq$ 2) at 12 months after randomization;
3. Proportion of patients with no or minimal disability (mRS $\leq$ 1) at 3 months and 12 months after randomization;
4. Change in NIHSS score at 3 months after randomization;
5. Proportion of composite cerebrovascular events (ischemic stroke, intracerebral hemorrhage, myocardial infarction, and vascular death) at 3 months and 12 months after randomization;
6. Difference of EuroQoL Group 5-Dimension (EQ-5D) score at 3 months and 12 months after randomization;
7. Change in Barthel Index (BI) score at 3 months and 12 months after randomization;
8. Difference of platelet counts and coagulation indicators (prothrombin time, and activated partial thromboplastin time) at 3 months after randomization.

4.3 Primary Safety Outcome

Incidence of serious adverse events (SAEs) within 3 months of treatment.

4.4 Secondary Safety Outcomes

1. Incidence of symptomatic intracranial hemorrhage within 3 months of treatment;
2. Incidence of all-cause mortality within 3 months of treatment;
3. Incidence of AEs within 3 months of treatment.

5. Study Design

5.1 Study Type

A randomized, double-blind, placebo-controlled, multi-center trial.

5.2 Randomization

Eligible patients will be randomized into an XST group and a control group at a ratio of 1: 1 to receive XST or placebo.

XST group:

XST, 60 mg/capsule, 120 mg per time, twice daily

Control group:

XST placebo, 60 mg/capsule, 120 mg per time, twice daily

6. Study Plan and Procedures

Study period: From November 2017 to December 2021;

Duration of treatment: 3 months;

Study period for patients included in the trial: 12 months;

The endpoint of the whole trial is defined as the last visit of the last patient enrolled in the trial.

6.1 Visit Schedule

Measures	Randomization/Day 1	1 month	3 months	6 months	12 months
Demographic characteristics	X				
Presenting complaint	X				
Physical examinations	X	X	X		
mRS	X	X	X	X	X
Past medical history	X				
NIHSS	X	X	X		
Head CT/MRI	X				
ASPECTS	X				
Laboratory tests	X		X		
ECG	X				
Verifying inclusion/exclusion criteria	X				
Signed written informed consent	X				
Randomization	X				

Measures	Randomization/Day 1	1 month	3 months	6 months	12 months
Compliance		X	X		
TOAST classification	X				
EQ-5D scale	X	X	X	X	X
BI	X	X	X	X	X
Concomitant medications		X	X	X	X
Capsule administration	X	X	X		
AEs/SAEs		X	X	X	X

6.1.1 Visit 1: Screening/Randomization Day (Day 1)

- Signed written informed consent;
- Demographic characteristics;
- Relevant medical history and surgical history;
- Vital signs and physical examination;
- Head CT/MRI;
- Trial of Org 10172 in Acute Stroke Treatment classification;
- 12-lead electrocardiograph (ECG);
- Alberta Stroke Program Early CT Score, NIHSS, mRS, EQ-5D , and BI scores;
- Laboratory tests: hematology (including white blood cell count, neutrophil count, hemoglobin, and platelet count), urinalysis (including white blood cell, ketone bodies, and occult blood), coagulation (including prothrombin time, and activated partial thromboplastin time), hepatic and renal functions (including blood creatinine, and serum aminotransferase), electrolyte (including K^+, and Na^+), fasting serum glucose, and lipid (triglyceride, and total cholesterol);
- Current medications;
- Patients will be advised to start the corresponding treatment on the day when the patient meets the inclusion criteria. If the patient does not meet the inclusion criteria, the reason for the exclusion will be recorded in Patient Screening Records;
- Drug distribution.

The test results obtained at Visit 1 will be used as the baseline values;

6.1.2 Visit 2: 1 Month after Randomization, Face-To-Face Follow-Up (Month 1 ± 3 days)

- Vital signs and physical examination;
- NIHSS, mRS, EQ-5D, and BI scores;
- AEs and SAEs;
- Medication adherence;

- Current medications;
- Cardiovascular and cerebrovascular events: new strokes (ischemic stroke and intracerebral hemorrhage), and new composite cerebrovascular events (ischemic stroke, intracerebral hemorrhage, myocardial infarction, and vascular death).

6.1.3 Visit 3: 3 Months after Randomization, Face-To-Face Follow-Up (Month 3 ± 7 days)

- Vital signs and physical examination;
- NIHSS, mRS, EQ-5D, and BI scores;
- Laboratory tests: hematology (including white blood cell count, neutrophil count, hemoglobin, and platelet count), coagulation (prothrombin time and activated partial thromboplastin time), hepatic and renal functions (blood creatinine and serum aminotransferase);
- AEs and SAEs;
- Medication adherence;
- Current medications;
- Cardiovascular and cerebrovascular events: new strokes (ischemic stroke and intracerebral hemorrhage), and new composite cerebrovascular events (ischemic stroke, intracerebral hemorrhage, myocardial infarction, and vascular death).

6.1.4 Visit 4: 6 Months after Randomization, Face-To-Face or Telephone Call Follow-Up (Month 6 ± 7 days)

- mRS, EQ-5D, and BI scores;
- AEs and SAEs;
- Current medications;
- Cardiovascular and cerebrovascular events: new strokes (ischemic stroke and intracerebral hemorrhage), and new composite cerebrovascular events (ischemic stroke, intracerebral hemorrhage, myocardial infarction, and vascular death).

6.1.5 Visit 5: 12 Months after Randomization, Face-To-Face or Telephone Call Follow-Up (Month 12 ± 7 days)

- mRS, EQ-5D, and BI scores;
- AEs, SAEs, and endpoints;
- Current medications;
- Cardiovascular and cerebrovascular events: new strokes (ischemic stroke and intracerebral hemorrhage), and new composite cerebrovascular events (ischemic stroke, intracerebral hemorrhage, myocardial infarction, and vascular death).

6.2 Protocol Compliance

6.2.1 Withdrawal Determined by the Investigator

1. Patients undergoing allergic reactions or SAEs will withdraw from the study as judged by the physician;
2. The patient's symptoms worsened after medication, and in order to protect the patient, the patient was withdrawn from the study and received other effective treatment;
3. Patients experience some comorbidities, complications, or special physiological changes during the study, affecting the efficacy and safety evaluations;

4. Patients with poor compliance during the study, i.e., underdosing by less than 70% or overdosing by more than 130% of the required doses;

5. Patients who are midway breaking the blindness during the study for any reasons;

6. Patients who are considered not suitable to continue the study as judged by the investigator.

6.2.2 Self-withdrawal by Participants

The Informed Consent Form (ICF) provides that the patient is entitled to withdraw from the study, or the patient may also be lost to follow-up as no longer receive the drug or tests even though he/she has not clearly requested to withdraw (classified as withdrawal or dropout).

6.2.3 Elimination Criteria

Patients who should not have been enrolled or who have completed the study but violated some of the requirements of the protocol during the study will be eliminated as judged by investigators, including patients who:

1. Have not taken the drug;

2. Have no test records after drug administration;

3. With unavailable efficacy and safety evaluations due to the administration of a certain prohibited drug.

7. Treatment

7.1 Identification of IMPs

7.1.1 IMPs

Xuesaitong soft capsules; Specification: 0.33 g/capsule (60 mg panax notoginseng saponins per capsule); Dosage form: soft capsule;

Manufacturer: China Resources Kunming Shenghuo Pharmaceutical Co., Ltd.

Xuesaitong soft capsules placebo; Specification: 0.33g/capsule; Dosage form: soft capsule;

Manufacturer: China Resources Kunming Shenghuo Pharmaceutical Co., Ltd.

7.1.2 Packaging of IMPs

The drug products will be packed into a big box for each patient, i.e., Xuesaitong soft capsules/Xuesaitong soft capsules placebo. Each center will prepare adequate drugs. Each patient will be designated a randomization number corresponding to a certain box of drugs. The name of the IMPs, usage and dosage, storage conditions, production batch numbers, production date, and duration of use or expiry dates, as well as the notice "For clinical study use only" will be clearly marked on each package and label of drugs.

Each big box will contain a medium box and a small box, among which the small one will be used from day at randomization to 1 month after randomization, and the medium one is for 1 to 3 months after randomization.

A small box will include XST or XST placebo, with a specification of 0.33 g/capsule, 12 capsules/plate, and 2 plates/box, with a total of 6 boxes.

A medium box will include XST or XST placebo, with a specification of 0.33 g/capsule, 12 capsules/plate, and 2 plates/box, with a total of 12 boxes.

Labels for small boxes

**Xuesaitong soft capsules for clinical study<br>(For clinical study use only)**	
Packaging:	6 boxes of Xuesaitong soft capsules or Xuesaitong soft capsules placebo

Usage and dosage:	Oral, Xuesaitong soft capsules, 60 mg/capsule, 120 mg per time, twice daily
Storage:	Stored in a cool place (not more than 20 °C) under shade and tightly closed
Batch no.:	Xuesaitong soft capsules/placebo:
Production date:	Xuesaitong soft capsules/placebo:
Expiration date:	December 2021
Note:	Please consult doctors if you have any questions
	Unused drugs will be uniformly recycled
Supplied by:	China Resources Kunming Shenghuo Pharmaceutical Co., Ltd.

Labels for medium boxes

**Xuesaitong soft capsules for clinical study**	
**(For clinical study use only)**	
Packaging:	12 boxes of Xuesaitong soft capsules or Xuesaitong soft capsules placebo
Usage and dosage:	Oral, Xuesaitong soft capsules, 60 mg/capsule, 120 mg per time, twice daily
Storage:	Stored in a cool place (not more than 20 °C) under shade and tightly closed
Batch no.:	Xuesaitong soft capsules/placebo:
Production date:	Xuesaitong soft capsules/placebo:
Expiration date:	December 2021
Note:	Please consult doctors if you have any questions.
	Unused drugs will be uniformly recycled.
Supplied by:	China Resources Kunming Shenghuo Pharmaceutical Co., Ltd.

Labels for big boxes

Packaging:	1 small box and 1 medium box
Usage and dosage:	Oral, Xuesaitong soft capsules, 60 mg/capsule, 120 mg per time, twice daily
Storage:	Stored in a cool place (not more than 20 °C) under shade and tightly closed
Batch no.:	Xuesaitong soft capsules/placebo:
Production date:	Xuesaitong soft capsules/placebo:
Expiration date:	December 2021
Note:	Please consult doctors if you have any questions.
	Unused drugs will be uniformly recycled.
Supplied by:	China Resources Kunming Shenghuo Pharmaceutical Co., Ltd.

7.2 IMP Records

The commissioning unit will provide all the investigational medicinal drugs, these drugs will be stored in a safe place under the required conditions, and be accessible only to the personnel involved in the study.

Investigators and/or pharmacists will maintain records of drug deliveries to the centers, list of delivered drugs, drug distributing and returning records, and records of unused drugs recalled, or other disposed by the commissioning unit. These records will include the date, quantity, batch number/serial number, expiration date (if any), IMPs, and unique number of the patient in the trial. Investigators will keep the records to confirm that patients have received the IMPs in accordance with the study protocol and all drugs from the commissioning unit have been used for the intended purpose. At the end of the trial, remaining drugs will be returned to the commissioning unit; investigators will ensure that patients returned all unused or remaining drugs, and investigators will not retain any of these drugs. The commissioning unit will count the remaining drugs after the trial and then eliminate them in accordance with relevant regulations in China, and send a copy of the elimination record to the principal investigator.

7.3 Storage of IMPs

All IMPs will be stored in safe place under appropriate conditions. Appropriate storage conditions are indicated on the IMP labels.

7.4 Concomitant Therapy

7.4.1 Contraindicated Concomitant Medications, Food Products, and Health Care Products

During the study (including the oral administration period of XST/XST placebo), the use of traditional Chinese medicinal products, food products, and health care products, containing panax notoginseng, will be disallowed. Using the following drugs, food products and health care products containing panax notoginseng constitutes a violation of the protocol, the IMPs will be discontinued in such cases and the reasons will be recorded, but the visit at Month 3 will be completed.

Traditional Chinese medicinal products containing panax notoginseng include: Xueshuantong capsules, Xueshuantong injections, Panax notoginseng saponins tablets, Panax notoginseng saponins capsules, Compound Danshen dripping pills, Sanqi Shangyao tablets, Sanqitongshu capsules, Sanqi Xueshangning capsules, and Yanghuo Sanqi capsules.

7.4.2 Allowed Concomitant Medications and Therapy

Medication will be administered by strictly following the "Chinese Guidelines for Diagnosis and Treatment of Acute Ischemic Stroke". In addition to the contraindicated concomitant drugs listed above, the use of other traditional Chinese medicinal products that stimulate blood circulation, remove stasis, and improve microcirculation will be avoided as much as possible; when necessary, no more than 2 of these drugs will be allowed. Other drugs can be selected reasonably for clinical use.

7.5 Treatment Compliance and Reliability

XST/XST placebo will be prescribed by a doctor and administered by a dedicated employee to the patient for keeping. Dedicated employees will be responsible for managing the IMPs in the study center, including receiving, distributing, counting, and recycling. Investigators will ensure that IMPs are stored in a safe, separate, and locked place in the study center under the conditions meeting the storage requirements of the IMPs. No one will be allowed to access the drugs without the consent of the investigator. Empty packaging boxes will be stored and managed in the same way as the IMPs. The compliance of the patient will be evaluated by calculating the number of drugs returned at each follow-up. Usage of all drugs (including IMPs) will be recorded in the medical record and the corresponding position of the Case Report Form (CRF). Discontinuation dates of IMPs will be recorded. Concomitant medications will be recorded at each visit.

7.6 Discontinuation of IMPs

IMPs will be administered as continuously as possible, but patients may discontinue the study treatment and evaluation at any time. When an IMP is discontinued, a decision for allowing temporary discontinuation is reached, if not, permanent discontinuation will be the last resort. Any drug discontinuation will be completely recorded on the corresponding page of the CRF. Patients will participate in the trial as long as possible under any circumstances. Pregnancy will lead to the permanent discontinuation of the trial under any circumstances.

7.6.1 Temporary Discontinuation of Study Treatment

Medication of the IMPs will be resumed once the investigator believes that the IMP is likely unrelated to the event onset based on his/her best clinical judgment and there are no other contraindications against the continuation of the study. Resumed medications will be done under a close and reasonable monitoring. Suspension time of the study will be recorded in detail if it is determined that the study can be restarted.

7.6.2 Permanent Discontinuation of Study Treatment

1. Decided by patients. Patients may terminate the treatment freely at any time without affecting their further treatment.
2. Decided by investigators. Including but not limited to the following:
    1) Mistaken enrollment that does not meet the Inclusion Criteria/Exclusion criteria, and will put the patient at a higher risk;
    2) AE is considered to be related to the study treatment and the investigator believes that continuing treatment may pose a higher risk;
    3) Poor compliance with the protocol;
    4) Pregnancy.

Discontinuation of IMPs does not mean discontinuation of follow-up or withdrawal from study. The study evaluation and telephone follow-up for all patients will be continued after the drug discontinuation.

7.6.3 Procedure of IMP Discontinuation for Patients

Patients who have permanently discontinued the IMPs will receive conventional therapies, and, if applicable, continue the regular visits as follows: Patients who have decided to discontinue the IMPs will be asked for reasons and whether they have experienced any AE. This information will be verified and recorded by investigators in the corresponding positions in the CRF. Information of all patients will be collected as much as possible during the whole study period, especially all the potential endpoint events. The complete withdrawal from study (withdrawal of the informed consent) may have a direct impact on the potential validity of the data, so it will be avoided as much as possible. Patients may continue to participate in the study according to the various following options if the patient has permanently discontinued the administration of the IMPs before the end of the study:

1. Patient agrees to participate in discontinuation visits and continues to attend visits in person.

This is the most ideal option, but the consent from the patients toward treatment discontinuation will be obtained before implementation. If the patient agrees, he/she will receive a premature treatment discontinuation visit (PTDV) on the day of discontinuation. After PTDV, the patient will also participate in the subsequent visits as scheduled, and data collection will be continued as per the protocol.

2. Patient refuses to attend visits in person but accepts to receive the follow-up by other means.

If the patient refuses to attend visits in person but accepts to receive the follow-up by other means, he/she will receive PTDV on the day of discontinuation. The subsequent visits before the end of the study will be conducted by other means (regular phone calls, and contact at the end of the study), which are applicable to patients who have not withdrawn the informed consent or withdrawn from the study.

3. Patient rejects follow-up by any means.

The patient rejects follow-up by any means, that is, the patient formally withdraws from the study and withdraws the informed consent. This decision will be recorded, and when the double-blind study period scheduled for such patients ends (3 months after the randomization), the survival status information of the patient will be collected via public channels pursuant to local laws. This plan will be avoided as much as possible.

7.6.4 End of Treatment

Three months after the randomization, IMP treatment will end, and the attending physician of the patient will determine the subsequent treatment regimens.

7.6.5 End of Follow-up

After the end of the study, patients will be required to receive the follow-up as scheduled. The investigator will complete the last visit while collecting the data of outcomes of efficacy and safety, AEs/SAEs, concomitant medications, mRS, EQ-5D, and BI scores.

7.7 Withdrawal from the Study

Without affecting their rights to acquire further treatment, patients will be allowed to withdraw from the study at any time, for example, by permanently discontinuing IMPs or withdrawing from the evaluation (withdrawal of informed consent). The investigator will certify the withdrawal of informed consent from this study and record it on the CRF and ICF. The ICF will, if possible, be re-signed with the signing date by the patient and the investigator. Any patient withdrawing from the study will be questioned about the reason for the withdrawal and inquired whether he/she has experienced any AE. The CRF will include the cause of the permanent discontinuation of the IMP treatment as well as the administration date of the last dose.

Patients who have permanently discontinued the IMPs will receive conventional therapies and will be expected to continue receiving the visits if applicable. One of the options "A" to "C" will be adopted if a patient declines all future follow-up visits of the study and formally withdraws the informed consent:

A. If possible, a patient receives a PTDV when the he/she discontinues the treatment and withdraws the informed consent of continued evaluation;

B. If the patient disagrees to option "A", which will be documented, a PTDV by other means (for example, via phone call) will be arranged and recorded in the CRF and ICF;

C. If the patient disagrees to both options "A" and "B", the disagreement will be recorded in the CRF and ICF.

It is crucial to collect as much information as possible during the study, especially survival statuses (death or survival), to ensure the validity of the study data. Therefore, at the end-of-treatment visit and end-of-study visit, investigators will make an effort to gather information on the survival statuses of all patients who have withdrawn their informed consent from public data sources. Patients who have withdrawn from the study will not be replaced.

8. Study Management

8.1 Enrollment and Randomization of Patients

Investigator will obtain the ICF signed by the patient or his/her legal representative before the randomization and certify the eligibility of the patient.

8.2 Randomization Procedures

For patients eligible for randomization, each center will assign randomization codes in strict sequential order. Randomization codes will be generated by SAS 9.4 (SAS Institute, Inc, Cary, NC) through block randomization to achieve balance in the allocation of patients between the two groups (1:1) as much as possible. The number of randomized patients planned in this study is approximately 3000.

At the Visit 1, the investigator will provide the randomization codes of patients to the dedicated drug-distributing employees in the sequence of patient enrollment, and the employees will identify the unique identification number on the drug box for drug supply at Visit 1. Patients will be required to complete the first administration of the IMPs within 24 hours after randomization.

8.3 Procedures of Mistakenly Included or Randomized Patients

By no means would a patient who violates the Inclusion Criteria/meets the Exclusion criteria be included in this study or randomized. This principle will be adhered to under any circumstances.

Patients will be treated by the following procedures if he/she violates the Inclusion Criteria/meets the Exclusion criteria but is already enrolled in this study:

1. The investigator or Clinical Research Associate (CRA) will immediately inform the physician of the study team and guarantee the safety of the patient as the priority;

2. The study treatment will be stopped if continued treatment may endanger the patients. The decision whether to discontinue the IMPs will be made after a discussion between the physicians and investigators of the study team. The reasons for discontinuing the treatment will be clearly recorded. The patient will receive follow-up visits according to the prescribed procedure until the end of the study, which is consistent with the intention-to-treat principle.

3. The reason for continuing the study will be clearly recorded if it is believed that the continued treatment will neither endanger the patient nor affect the therapy of the disease. The patient will keep receiving follow-up visits according to the prescribed procedure.

8.4 Blinding and Unblinding Procedures

8.4.1 Methods to Ensure Blindness

To ensure the implementation of the blinding method, the personnel involved in randomization coding or who may know the codes, and the doctors or nurses involved in drug management will not participate in the selection

of patients, evaluation of efficacy and safety of drug to avoid bias. In addition, to ensure the blindness, the drug appearance, packages, and batch numbers in the two groups will be identical. Each box of drugs will be marked with a unique number. This number will be used to designate the treatment the patient receives, but will not inform the investigator or the patient about the treatment assignment. Data and Safety Monitoring Board (DSMB) will regularly review the efficacy and safety data, including the incidence of AEs and results of safety outcomes, to ensure the safety of patients. During the study, basic safety and efficacy data will be provided to the DSMB by an independent statistician.

8.4.2 Emergency Unblinding Method

Emergency unblinding cards are available to investigators or pharmacists to obtain individual treatment codes, which represent the treatment that each randomized patient receives. For specific routine procedures, please refer to the operation manual provided to each study center. The code and randomization information will be disclosed only when the patient is in a medical emergency to ensure the patient will be treated appropriately. Investigators will report to the sponsor and the principal investigator of the leading institute before unblinding. If all blinding codes are leaked or the emergency unblinding rate exceeds 20%, the double-blind trial will be invalid.

8.5 Blind Review

After the blindness is reviewed and the established database is confirmed correct, the principal investigator and the statistical analysts will lock the data. The locked data file will not be changed.

9. Quality Control (QC)

During the clinical trial, the CRA will regularly visit the study center for monitoring to ensure the protocol is implemented compliantly, and review the source data to ensure the consistency with the data on CRF. Employees involved in the clinical trial will receive uniform training.

Investigators will prudently fill in each item of the CRF as required with accurate and detailed information to ensure the accuracy and reliability of the CRF data. All the observation results and findings in the clinical trial will be verified to ensure data reliability and that the conclusions of the clinical trial are completely based on source data.

9.1 Storage and Auditing of Source Data/Documents

The source documents represent the basis for the real existence of the patients and the reliability of the collected data, and will be kept in each study center. The data in the CRF is from source documents and will be consistent with source documents; if not, any deviation will be explained. The following items recorded in the CRF will come from source documents:

- Basic information of patients (name abbreviation, gender, birthday, height, and weight);
- Date of patients participating in the trial;
- Follow-up dates of patients;
- Medical history (concomitant diseases, start and end date, and changes);
- Medication history (concomitant therapies and medications, start and end dates, and changes);
- AEs (onset and end dates, and changes);
- SAEs (onset and end dates, and changes);
- Laboratory test results;
- ECG results and the test results by other medical instruments.

All other information will be directly recorded in the CRF and treated as source data. Investigators will accept

the sponsor's monitoring and auditing, ethics committee's review, and relevant regulatory authorities' inspections, and allow the employees from the above parties to refer to all the source data/documents. All source data, including the copies of the results of laboratory tests and other medical tests, will always be ready for the review of the CRA designated by the sponsor and the CFDA (now NMPA). The CRA will check all CRF and ICF and verify the accuracy of the data in these documents.

9.2 Pre-study Task

Before the first patient is enrolled, the sponsor's representative will visit the study center to:

1. Confirm whether the study center's facilities are adequate;
2. Confirm whether there are suitable patients to be enrolled in the study center;
3. Discuss with investigators (and other study-related personnel) their responsibilities for the protocol and the responsibilities of the sponsor or its representative.

9.3 Training of Study Center Personnel

Before the first patient is enrolled in the study, the undertaking unit will provide standardized trainings and examinations to the principal investigator and all other investigators of the sub-center, and the study will only be started in the sub-center once the examinations are passed. The principal investigator will make sure that all study-related personnel are trained properly for the study with complete training records.

9.4 Study Monitoring

During the study, the sponsor will contact the study centers regularly, including:

1. Visits to provide information and support to the investigators;
2. Confirm that the facility still meets the requirements;
3. Confirm whether all study-related personnel follow the protocol, the data is recorded in the CRF promptly and accurately, and numbers of the IMPs are checked;
4. Compare the data in the CRF with the records of the patients generated in the study centers, including the inspection on ICF of the patient. All original records of each patient will be directly examined.

Investigators will contact the sponsor during the follow-up intervals for information and recommendations related to the study.

9.5 Source Data

The undertaking unit will store all original records and source data, which will be inspected and reviewed by the CFDA (now NMPA).

9.6 Study Progress

If the procedures at a study center are not conducted in accordance with Good Clinical Practice (GCP) requirements, or if the recruitment period is too long, the study at that center may be terminated. The study sponsor may terminate the study early due to safety concerns about the study.

10. AEs

Any information on AEs, whether mentioned by patients and found by investigators, or detected in physical examinations, laboratory tests, or by other means, will be recorded in the AE pages of the CRF, and these AEs will be treated and reported pursuant to related laws.

10.1 Definition of AEs

Any adverse medical events, or worsening of pre-existing medical events from the start of the randomization

to the last visit, whether or not causally related to the IMPs, are regarded as AEs. During the clinical study, any unexpected events that happen to the patients will be defined as AEs. Adverse medical events include clinical symptoms (such as nausea and chest pain), signs (such as tachycardia and hepatomegaly), and abnormal test results (including laboratory tests and ECG). The physicians will report all AEs directly observed by themselves or spontaneously reported by the patients concisely with medical terminology. Adverse drug reactions are the reactions that are directly or indirectly related to drugs. To prevent any omission of adverse drug reactions when the relationship between an AE and drugs can hardly be determined, all AEs will be recorded in the CRF, including occurrence time, severity, duration, treatment measures, and outcome. Additionally, all relationships between AE and drugs will each be stated.

10.2 Observation and Records of AEs

All patients who have received at least 1 dose of the IMPs will undergo a safety evaluation with the following events recorded: AEs, SAEs, deaths, recurrent strokes, drug discontinuation for any reasons, abnormal laboratory test results, vital sign changes, and changes to physical examination results. Any AE that happens from the start of the randomization to 12 months after the enrollment or the last visit will be recorded. A patient with an AE will be followed up until the event disappears, restored to the baseline level, or becomes clinically insignificant. All AEs occurring during the study will be recorded in the CRF. The evaluation will include the name of AE, severity, outcome, relationship between AEs and IMPs, and treatment measures.

10.3 Severity of AEs

1. Mild: mild symptoms or diseases that improve rapidly after drug discontinuation and require no treatment;
2. Moderate: transient damages that do not require hospitalization or prolonged hospitalization but do require treatment or interventions, and can be easily relieved;
3. Severe: transient damages that require an out-patient to be hospitalized or an in-patient to prolong the hospitalization by more than 7 days, or permanent damages to systems/organs, or life-threatening events (asphyxia, shock, coma, and other symptoms that require first aid treatment).

10.4 Correlation between the AE and Drugs

Investigators will evaluate the correlation between AEs and the IMPs as well as concomitant medications:

1. Definitely related: the onset time follows the time sequence after administration, and the AE is consistent with a known type of adverse reactions of the suspected drug; the adverse reaction improves or disappears after the dose reduction or drug discontinuation but reappears after resuming the drug administration;
2. Probably related: the onset time follows the time sequence after administration, and the AE is consistent with a known type of adverse reactions of the suspected drug; the adverse reaction improves or disappears after the dose reduction or drug discontinuation but it may also be caused by the clinical status of the patient or other reasons;
3. Possibly related: the onset time follows the time sequence after administration, and the AE is consistent with a known type of adverse reactions of the suspected drug; the adverse reaction improves or disappears after the dose reduction or drug discontinuation but it can be explained by the clinical status of the patient or other reasons;
4. Unlikely related: the onset time is unlikely to follow the time sequence after administration, and the AE is hardly consistent with a known type of adverse reactions of the suspected drug; the adverse reaction is possibly caused by the clinical status of the patient or other reasons;
5. Definitely unrelated: the onset time does not follow the time sequence after administration, and the AE is hardly consistent with a known type of adverse reactions of the suspected drug; the reaction can be explained by the clinical status or other reasons of the patient and it improves or disappears after removing the clinical status or other reasons;
6. Unknown.

Summarize the AEs of the first three criteria in each CRF and calculate the incidence of drug-related AEs.

10.5 SAEs

SAEs are defined as the AEs leading to following results:

1. Death;
2. Life-threatening (the AE occurrence leads to a life-threatening risk to the patient, excluding the AEs that are assumed to be potential causes of deaths if worsened) event;
3. Hospitalization or prolonged hospital stay;
4. Continuous or significant disability/dysfunction;
5. Congenital malformations or birth defects;
6. Medical events considered as SAEs by the investigator.

In case of a SAE during the trial, whether related to the IMPs or not, the investigator will immediately take treatment measures for the patient for their safety and will report it to the CRA, the ethics committee of the center, and the undertaking unit within the 24 h after it occurs. The undertaking unit will report it to ethics committees and the sponsor, as well as the CFDA (now NMPA) for filing. The investigator will complete the filling of the SAE form.

In case of a SAE that has not been resolved after the end of the trial or the early withdrawal of the patient will be followed up until any of the following circumstances:

1. Event disappears or is resolved;
2. Stable event;
3. Event is restored to baseline;
4. Event is relieved to be clinically insignificant;
5. Event can be attributed to the drugs other than the IMPs or factors unrelated to the study, or that any more information is unlikely to be obtained (the patients or medical staff refuse to provide more information, or there is evidence that the patient is still lost to follow-up despite best efforts).

10.6 Record, Management, and Report

- Any AEs during the trial, whether related to the IMPs or not, will be documented;
- AEs related to IMPs will be managed carefully;
- Patients will be required to report any changes after the drug administration;
- Investigators will avoid asking inductive questions. While observing the efficacy, the patient will be closely monitored for AEs and unexpected toxic or side effects (including symptoms, signs, and laboratory test results), which will be analyzed for reasons, judged, followed up, and recorded;
- Incidence of AEs will be calculated;
- Symptoms, severity, occurrence time, duration, treatment measures, outcomes, correlation to drugs, and follow-up modes of the AEs during the trial will be recorded in CRF in detail, and the CRF will be signed and dated;
- When an AE is found, investigator may decide whether to discontinue the study according to the patient's condition, and will carry out a follow-up investigation on the case of drug discontinuation due to AEs and record the process and results in detail;
- In case of any SAE or important AE occurring during the study, the investigator will initiate emergency procedures;

- Among all patients who have received the study treatment, those experiencing AEs will be included in AE statistics, regardless of whether they have completed the whole trial.

10.7 Emergency Procedures

In case of any SAE or important AE occurring during the study, the investigator will immediately organize a rescue for the patient to ensure his/her safety. The undertaking unit will be informed of the event immediately via telephone/fax, to clarify whether or not the event is IMP related or if the IMP has been administered. Subsequently, the CRA will provide a written report to describe the details and outcome of the event. In addition to confirmation of the content reported by telephone/fax, the written report will also contain other unmentioned information. The CRA will check the written report and report to the drug safety department according to GCP standard operating procedures within 24 h or no later than the second working day after receiving the phone call.

11. Ethical and Regulatory Requirements

11.1 Independent Ethics Committee Approval

According to the local regulations, all protocols (and relevant amendments, if any), including patient information and informed consent, will obtain the approval of independent ethics committee before implementation. The reports related to the progress of trial, including notification of matters regarding the completion or termination of trial, will be submitted to the independent ethics committee by the investigator or sponsor.

11.2 Patient Information and Informed Consent

These documents will be created according to International Conference on Harmonization-Good Clinical Practice guidelines, and will be provided to the independent ethics committee as separate documents. Only approved versions will be used for the trial. During the process to obtain and record informed consent, the investigator will comply with relevant local regulatory requirements and the Declaration of Helsinki. Prior to initiation of activities related to the study, investigator will verbally describe the information on this study to the patients, and provide relevant written information that is readable and understandable for the patients. The ICFs signed and dated voluntarily by each patient will be obtained. The written ICF will be signed and dated by the personnel who is responsible for the implementation of informed consent procedures. The patients enrolled in the study will be informed that they can refuse to participate in the study and their medical care will not be affected. Information on their medical conditions will be collected and recorded, and all findings will be handled in strict confidence. Patients will be provided with the copies of patient information and ICF.

11.3 Essential Documents

1. Latest curriculum vitae of the principal investigator;
2. Investigator signature page in the protocol;
3. Copies of dated documents related to the protocol and local investigators that have been approved by the independent ethics committee, including any amendments, patient information and ICF, compensation (if any), and any other documents that have been submitted;
4. Contracts signed between the sponsor and the study centers;
5. Approval documents from the local hospital management authority if necessary;
6. Other relevant documents that are required before the initiation of study.

12. Data Management

12.1 Data Acquisition and CRF

CRFs will be filled in by the investigator and will be completed for each enrolled patient. An electronic data

capture system will be used for data acquisition for all CRFs. All data will be reviewed and submitted by the CRA to the data administrator for data management.

12.2 Data Management Procedures and Tasks

12.2.1 Design of CRF and Database

The design of CRF will ensure that the collection of all data meets the requirements for statistical analysis as specified in the protocol. Guidelines for completing the corresponding CRFs will be established. Logical verification will be established according to the design description of database, and the database will be put into use after passing the user acceptance testing.

12.2.2 Receipt of Data

Data collection and management of this study will follow the requirements of GCP. The data administrator designated by the statistics agency will be responsible for data acquisition and management. The data administrator will compile a program for data acquisition and management. The clinical investigator or clinical research coordinator will fill in the CRFs in an accurate, timely, complete, and standardized manner according to the CRF guidance. Instructions for data acquisition will be formulated before data acquisition to determine relevant requirements and methods. For any question about the CRF, the data administrator will create a data rating questionnaire, and send a query to the investigator through the CRA. The investigator will give a reply as soon as possible, and the data administrator will modify and confirm the data according to the investigator's reply, or send another questionnaire when necessary.

12.2.3 Data Validation and Query

The data administrator, CRA, medical personnel, and statistician will perform data validation according to the content, method, and validation requirements as stated in the data validation plan.

12.2.4 Medical Coding

The coding standards, procedures, and dictionary will be established to match the descriptions of AE, diagnosis, concomitant medications, previous medications, and past medical history collected from the CRF using International Classification of Diseases-10 and WHO Anatomical Therapeutic Classification.

12.2.5 Database Locking, Unlocking, and Re-locking

Formulate the responsible person and standard operating procedures to be executed for database lock, and specify the conditions and procedures for unlocking and re-locking the database after locking.

12.2.6 Data Export and Transfer

Describe the file formats for data export and transfer, export content (database, variable name, and variable value code), submission procedures, and transmission medium. The transmission medium will conform to national regulations and regulatory requirements.

12.2.7 Archiving Requirements for Data and Data Management Documents

Data and the time of their input/import into the database, recorders, data audit trail, and documents generated during data management will all be kept intact. Data generated during data management typically include, but is not limited to: clinical study data, external data, database metadata, reference value range for laboratory tests, change control list of logical verification and derived data, data query form, and procedure code. Documents generated during data management typically include, but are not limited to: data management plan, blank CRF, CRF completion guidance, completed CRF in portable document format, annotated CRF, database design description, instructions for data entry into the database, data validation plan, data QC, and validation reports.

12.3 Data Quality Management

The data management plan will determine the QC items, QC methods (e.g., QC frequency, sample selection method, and sample size), quality requirements and specifications, and remedial measures for failure to meet the expected specifications during data operation and management.

13. Statistical Analysis and Sample Size

All statistical analyses will be carried out using the SAS 9.4 (SAS Institute, Inc, Cary, NC) software according to the predetermined statistical analysis plan. Comparisons between two groups are performed using a two-side test ($\alpha = 0.05$). A difference of $p \leq 0.05$ is considered to be statistically significant.

13.1 Analysis Cohorts

13.1.1 Modified Intention-To-Treat Cohort

According to the basic principle of intention-to-treat analysis, all patients after randomization who received at least one record for drug administration and efficacy evaluation will be included in the modified intention-to-treat cohort. During analysis with the modified intention-to-treat cohort, missing values will be estimated using the last observation carried forward method. The modified intention-to-treat population will be the primary population for efficacy evaluation in this study.

13.1.2 Per-Protocol Cohort

Per-protocol cohort will include all patients who have completed the treatment prescribed in the protocol or those without major violations of the trial protocol. The exact definition of major protocol violation will be finalized during data review, generally including, but not limited to the following conditions: failure to meet the primary inclusion criteria, treatment that seriously interferes with efficacy evaluation after enrollment, poor compliance, and follow-up that seriously exceeds the time window. Per-protocol population will be the secondary population for efficacy evaluation.

13.1.3 Safety Cohort

Safety cohort is defined as a collection of all patients who have received at least one dose of drug treatment.

13.2 Statistical Analysis Methods

13.2.1 Baseline Between-Group Comparisons

Wilcoxon rank-sum test will be used for measurement data. Chi-square test will be used for the comparability of enumeration data between the two groups.

13.2.2 Efficacy Analysis

Primary endpoint: According to the basic principle of intention-to-treat analysis, Chi-square test or Fisher's exact test will be used for data in the modified intention-to-treat cohort to evaluate efficacy. In addition, a logistic regression model will be adopted to calculate the odds ratio (OR) between the two treatment regimens, with center effects set as random effects in the model.

Secondary endpoint: The primary endpoint analysis strategy will be used for most secondary endpoint analyses. Wilcoxon rank-sum test will be used for measurement data to analyze differences between the two groups. For survival data, the Kaplan-Meier method will be used to estimate the survival rate of each group and plot survival curves, log-rank test will be used for efficacy evaluation, and Cox proportional hazards model will be used to calculate the hazard ratio (HR) between the two treatment regimens. For non-survival data, Chi-square test or Fisher's exact test will be used, and a logistic regression model will be used to calculate the OR value. Center effects will be set as random effects in all the models.

13.2.3 Safety Analysis

AEs and SAEs that have occurred will be listed separately and pooled for analysis. In each treatment group, the number and proportion of cases experiencing AEs will be pooled by system classification and items of interest. In addition, all deaths and SAEs will be described in detail in case narratives.

13.2.4 Interim Analysis

No interim analysis will be conducted in this study.

13.2.5 End-of-Study Follow-Up

Data collected from each group at the end of the study visit will be reported using descriptive statistics. The modified intention-to-treat cohort will be used for the analysis of efficacy outcomes, and the safety cohort will be used for the analysis of AEs.

13.3 Sample Size Determination

Based on previous findings, assuming that the proportion of patients in the control group with mRS ≤ 2 at 3 months is 45%, and the proportion in the XST group is relatively increased by 12%, with the significant level of 0.05 and the power of 0.80, 1342 patients are required in each group, and considering 10% lost to follow-up, 1492 patients are required in each group. Rounded to an integer, this trial is expected to enroll 3000 patients.

Power	Sample size of the XST group	Sample size of the control group	Response rate of IMP	Response rate of control drug	Difference in response rate between the two groups	Significant level
0.8000	2389	2389	0.4400	0.4000	1.100	0.0500
0.8000	1931	1931	0.4950	0.4500	1.100	0.0500
0.8001	1565	1565	0.5500	0.5000	1.100	0.0500
0.8001	1977	1977	0.4440	0.4000	1.110	0.0500
0.8002	1597	1597	0.4995	0.4500	1.110	0.0500
0.8002	1293	1293	0.5550	0.5000	1.110	0.0500
0.8001	1663	1663	0.4480	0.4000	1.120	0.0500
0.8001	1342	1342	0.5040	0.4500	1.120	0.0500
0.8004	1086	1086	0.5600	0.5000	1.120	0.0500
0.8002	1419	1419	0.4520	0.4000	1.130	0.0500
0.8001	1144	1144	0.5085	0.4500	1.130	0.0500
0.8001	924	924	0.5650	0.5000	1.130	0.0500
0.8002	1225	1225	0.4560	0.4000	1.140	0.0500
0.8003	987	987	0.5130	0.4500	1.140	0.0500
0.8001	796	796	0.5700	0.5000	1.140	0.0500
0.8002	1068	1068	0.4600	0.4000	1.150	0.0500
0.8003	860	860	0.5175	0.4500	1.150	0.0500
0.8002	693	693	0.5750	0.5000	1.150	0.0500

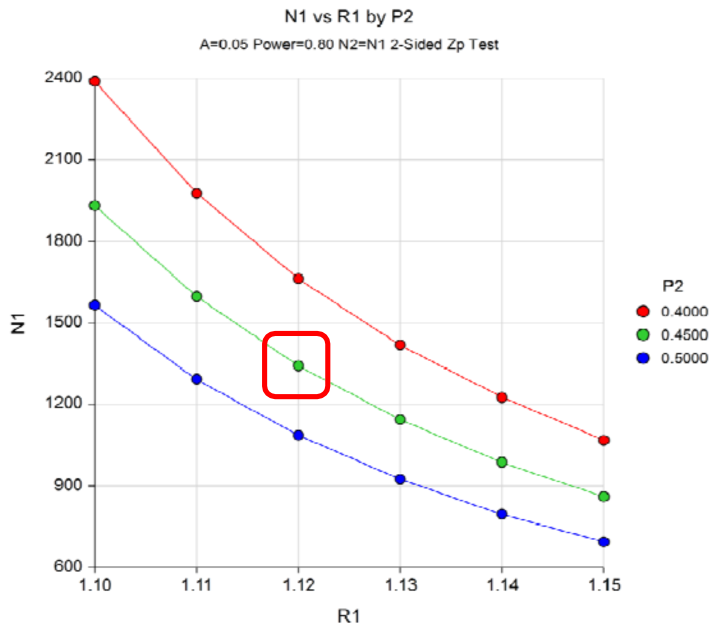

14. Principles for Protocol Amendment

All amendments to the protocol will be signed by all the signatories of the original protocol and recorded.

15. Principles and Criteria for Complete Termination of the Trial

- Serious safety issues occur;
- The drug is found to have a poor response or is even ineffective without any value;
- Major errors are found in the study protocol or major deviations occur during the implementation of the protocol, making it difficult to evaluate the drug effect;
- Discontinuation requested by the sponsor (due to cost or management issues);
- Authorization for the trial is revoked by the administrative authority.

16. Confidentiality

Personal medical information of patients obtained during the trial will be considered confidential and will not be disclosed to any third party except in the following circumstances:

1. Related medical information may be provided to the patient's physician in charge, or to other relevant physicians;
2. Data related to this trial may be provided to the following personnel and agencies for review, including participating physicians, sponsor's representative, ethics committees, and relevant regulatory authorities.

Further protection will be provided to patients via patient's identification code.

17. References

[1] Lozano R, Naghavi M, Foreman K, et al. Global and regional mortality from 235 causes of death for 20 age groups in 1990 and 2010: a systematic analysis for the Global Burden of Disease Study 2010 [J]. Lancet, 2012, 380(9859):2095-2128.

[2] Yang G, Wang Y, Zeng Y, et al. Rapid health transition in China, 1990-2010: findings from the Global Burden of Disease Study 2010 [J]. Lancet, 2013, 381(9882):1987-2015.

[3] Li Hao, Sun Honghui. Clinical Observation of Xuesaitong Injection in the Treatment of Intracerebral Hemorrhage [J]. Chinese Journal of Integrated Traditional and Western Medicine in Intensive and Critical Care, 2004, 11(1):50-52. [In Chinese]

[4] Liu Jianhui, Ji Fengyun, Wang Ting, et al. Experimental Study on the Protective Effects of Panax Notoginseng Saponins on Cerebral Ischemia-Reperfusion Injury [J]. Chinese Journal of Clinical Neurosciences, 2002, 10(1):90. [In Chinese]

[5] Cao Baowen, Du Yuebin. Effects of Panax Notoginseng Saponins on Hemorheology of Ischemic Cerebrovascular Diseases [J]. People's Military Surgeon, 2003, 46(12):695-697. [In Chinese]

[6] Li Shaoyan, Hu Jinmei, Zhang Shuning. Clinical Observation on the Safety and Effectiveness of Xuesaitong Soft Capsules in the Treatment of Convalescent Cerebral Infarction Involving Meridian and Collateral [J]. Hebei Medical Journal, 2010, 32(5):560-561. [In Chinese]

[7] Gao Linrong, Lan Hua. Xuesaitong Soft Capsules for the Treatment of 45 Cases of Convalescent Ischemic Stroke [J]. Chinese Medicine Modern Distance Education of China, 2012, 10(7):32-33. [In Chinese]

[8] Xie Xudong, Ji Fengyun, Wang Ting, et al. Efficacy Observation of Panax Notoginseng Saponins in the Treatment of 586 Cases of Acute Cerebral Infarction [J]. Hebei Medical Journal, 2001, 23(2):108-109. [In Chinese]

[9] Guo Ziqing, Liu Sixin, Chen Xuelian, et al. Efficacy Observation of Xuesaitong Soft Capsules in Combination with Ozagrel Sodium in the Treatment of 112 Cases of Acute Cerebral Infarction [J]. China Practical Medicine, 2011, 06(3):162-163. [In Chinese]

[10] Liu Yuanhong, Ge Xiaohang, Liang Jinhua, et al. Efficacy Observation of Xuesaitong Soft Capsules in the Treatment of 100 Cases of Acute Cerebral Infarction [J]. Yunnan Journal of Traditional Chinese Medicine and Materia Medica, 2005, 26(4):51. [In Chinese]

[11] Gao Na. Discussion on the Clinical Effects of Atorvastatin in Combination with Xuesaitong Soft Capsules on the Carotid Plaque Stability and Blood Lipid Level in Patients with Cerebral Infarction [J]. Chinese Journal of Practical Nervous Diseases, 2017, 20(6):93-95. [In Chinese]

[12] Tian Z, Pang H, Du S, et al. Effect of Panax notoginseng saponins on the pharmacokinetics of aspirin in rats [J]. Journal of Chromatography B Analytical Technologies in the Biomedical & Life Sciences, 2017, 1040:136-143.

[13] Gui Shuhua, Hu Lingling, Wang Tinggang, et al. Effects of Xuesaitong Soft Capsules in Combination with Aspirin on Carotid Plaque Stability and Body Antioxidant Function in the Treatment of Chronic Cerebral Infarction in the Elderly [J]. Guizhou Medical Journal, 2017, 10:1059-1061. [In Chinese]

849

Appendix 1. Diagnostic Criteria for Ischemic Stroke (Excerpts)

Diagnosis of ischemic stroke will be made according to the criteria set in the Fourth National Cerebrovascular Disease Conference of the Chinese Medical Association.

1. Atherosclerotic thrombotic cerebral infarction

1) Usually occurring at rest;
2) Mostly without obvious headache or vomiting;
3) Slow onset, typically progressive or periodic, usually related to cerebral atherosclerosis, and also seen in arteritis and hematological diseases;
4) Clear consciousness or mild disorder of consciousness generally 1-2 days after the onset;
5) Symptoms and signs of internal carotid artery system and/or vertebrobasilar system;
6) CT or MRI examination is required;
7) Lumbar puncture cerebrospinal fluid will normally contain no blood.

2. Cerebral embolism

1) Most cases have a sudden onset;
2) Most show no prodromal symptoms;
3) Clear consciousness or transient disorder of consciousness in general;
4) Symptoms and signs of internal carotid artery system and/or vertebrobasilar system;
5) Lumbar puncture cerebrospinal fluid will normally contain no blood, and hemorrhagic cerebral infarction may be considered if red blood cell is present;
6) Emboli can be classified into cardiogenic and non-cardiogenic emboli by source, and can be accompanied by embolism symptoms in other organs, skin, and mucous membranes.

3. Lacunar infarction

1) The disease is mostly due to arteriosclerosis in hypertension patients, characterized by acute or subacute onset;
2) Most cases have no disorders of consciousness;
3) CT or MRI examination is required to for clear diagnosis;
4) Clinical manifestations are not serious, commonly including pure sensory stroke, pure motor hemiplegia, ataxic hemiparesis, dysarthria-clumsy hand syndrome, or sensorimotor stroke;
5) Lumbar puncture cerebrospinal fluid will contain no red blood cells.

4. Asymptomatic infarction

This is a vascular disorder confirmed only by imaging for which no brain or retinal symptoms is documented, and the clinical diagnosis will be decided depending on the specific situation.

Appendix 2. The modified Rankin Scale

The mRS will be used to assess patients' functional outcome after stroke. Only symptoms developing after the stroke will be considered. If a patient is able to walk with the support of some assistive devices and requires no external assistance, then the patient will be considered able to walk independently. If two grades seem equally applicable to the patient, and further questions are unlikely to help make an absolutely correct choice, the more serious grade will be chosen.

**0-No symptoms at all**
Despite possible mild symptoms, the patient does not experience any new functional limitations or symptoms since the occurrence of stroke.
**1-No significant disability despite symptoms; able to carry out all usual duties and activities**
The patient experiences certain symptoms caused by stroke, no matter physically or cognitively (for example those affecting speaking, reading, or writing; physical movement; feeling; visual sense; swallowing; or emotion), but the patient is still able to engage in all the work and social and leisure activities that he/she could carry out prior to stroke. The key question used to distinguish grade 1 from grade 2 can be "Are there any things you did frequently in the past but can't do anymore after stroke?". Activities with a frequency more than once a month are considered "frequent".
**2-Slight disability; unable to perform all previous activities but able to look after own affairs without assistance**
After stroke, the patient is unable to perform some previous activities that he/she could complete before stroke (e.g., driving, dancing, reading, or working), but the patient is still able to look after him/herself without assistance. The patient can dress, walk, eat, go to the bathroom, prepare simple food, go shopping, and travel locally without the help from others. And the patient can live without supervision. It is assumed that patients at this grade can live alone at home for one week or above in the absence of caregiver.
**3-Moderate disability; requiring some help but able to walk without assistance**
Patients at this grade can independently walk (with or without the help of a walking aid), dress, go to the bathroom, and eat, but an assistance is required for more complex tasks. For example, shopping, cooking, or cleaning needs to be done by someone else, and visiting the patient more than once a week is necessary to ensure that these activities can be completed. Assistance is needed not just for physical care, but more for advice: for example, patients at this grade will need supervision or encouragement to deal with their finances.
**4-Moderately severe disability; unable to walk without assistance and unable to attend to own bodily needs without assistance**
The patient needs others' help with daily life, regardless of walking, dressing, going to the bathroom, or eating. The patient needs care for at least once a day, usually twice or more, or must live within close proximity of a caregiver. To distinguish grade 4 from grade 5, consider whether the patient is able to live alone normally during appropriate time spans within a day.
**5-Severe disability; bedridden, incontinent, and requiring constant nursing care and attention**
The patient needs to be taken care several times throughout the day and night, although no trained nurses are required.

6-Death.

Appendix 3. The National Institutes of Health Stroke Scale

A patient is scored according to the form and results are recorded. Scores given cannot be changed. Scores will reflect what the patient does, not what the clinician thinks the patient can do. The clinician will record answers while administering the exam and work quickly. Except where indicated, the patient will not be coached (i.e., repeated requests to patient to make a special effort). Detailed explanation will be provided in the form for items that are not assessed.

	Instructions	Score
1a	Level of consciousness (LOC):<br>The investigator must choose a response if a full evaluation is prevented by such obstacles (e.g., an endotracheal tube, language barrier, orotracheal trauma/bandages). A 3 is scored only if the patient makes no movement (other than reflexive posturing) in response to noxious stimulation.	0 = Alert; keenly responsive<br>1 = Not alert; but arousable by minor stimulation to obey, answer, or respond<br>2 = Not alert; requires repeated stimulation to attend, or is obtunded and requires strong or painful stimulation to make movements (not stereotyped)<br>3 = Responds only with reflex motor or autonomic effects or totally unresponsive, flaccid, and areflexic
1b	LOC questions:<br>(It is important that only the initial answer be graded and that the examiner not give any cues) The patient is asked the month and his/her age. The answer must be correct — there is no partial credit for being close. Aphasic and stuporous patients who do not comprehend the questions will score 2. Patients unable to speak because of endotracheal intubation, orotracheal trauma, severe dysarthria from any cause, language barrier, or any other problem not secondary to aphasia are given a 1.	0 = Answers both questions correctly<br>1 = Answers one question correctly<br>2 = Answers neither question correctly or unable to speak
1c	LOC commands:<br>The patient is asked to open and close the eyes and then to grip and release the non-paretic hand. Substitute another one step command if the hands cannot be used (extend tongue). Only the first attempt is scored. Credit is given if an unequivocal attempt is made but not completed due to weakness. If the patient does not respond to command, the task will be demonstrated to him or her (pantomime), and the result scored (i.e., follows none, one or two commands). Patients with trauma, amputation, or other physical impediments will be given suitable one-step command.	0 = Performs both tasks correctly<br>1 = Performs one task correctly<br>2 = Performs neither task correctly
2	Best gaze:<br>Only horizontal eye movements will be tested. Voluntary or reflexive (oculocephalic) eye movements will be scored, but caloric testing is not done. If the patient has a conjugate deviation of the eyes that can be overcome by voluntary or reflexive activity, the score will be 1. If a patient has an isolated peripheral nerve paresis (CN III, IV, or V), score a 1. Gaze is testable in all aphasic	0 = Normal<br>1 = Partial gaze palsy (gaze is abnormal in one or both eyes, but forced deviation or total gaze paresis is not present)<br>2 = Forced deviation, or total gaze paresis not overcome by the oculocephalic maneuver

	Instructions	Score
	patients. Patients with ocular trauma, bandages, pre-existing blindness, or other disorder of visual acuity or fields will be tested with reflexive movements, and a choice made by the investigator. Establishing eye contact and then moving about the patient from side to side will occasionally clarify the presence of a partial gaze palsy.	
3	Visual fields:<br>Visual fields (upper and lower quadrants) are tested using finger counting or visual threat, as appropriate. Patients may be encouraged, but if they look at the side of the moving fingers appropriately, this can be scored as normal. If there is unilateral blindness or enucleation, visual fields in the remaining eye are scored. Score 1 only if a clear-cut asymmetry, including quadrantanopia, is found. If patient is blind from any cause, score 3. Double simultaneous stimulation is performed at this point. If the patient is dying, score a 1, and the results are used to respond to item 11.	0 = No visual loss<br>1 = Partial hemianopia<br>2 = Complete hemianopia<br>3 = Bilateral hemianopia (blind including cortical blindness)
4	Facial palsy:<br>Ask — or use pantomime to encourage — the patient to show teeth or raise eyebrows and close eyes. Score symmetry of grimace in response to noxious stimuli in the poorly responsive or non-comprehending patient. If facial trauma/bandages, orotracheal tube, tape, or other physical barriers obscure the face, these will be removed to the extent possible.	0 = Normal<br>1 = Minor paralysis (flattened nasolabial fold, asymmetry on smiling)<br>2 = Partial paralysis (total or near-total paralysis of lower face, central paralysis)<br>3 = Complete paralysis (of one or both sides, absence of facial movement in the upper and lower face, peripheral paralysis)
5a	Left motor arm:<br>Extend the arm: 90 degrees if sitting or 45 degrees if supine. Drift is scored if the arm falls before 10 seconds. The aphasic patient is encouraged using urgency in the voice and pantomime, but not noxious stimulation. The assessor can lift the patient's arm to the required position and encourage the patient to hold on. Assess the affected side only.	0 = No drift; limb holds 90 (or 45) degrees for full 10 seconds<br>1 = Drift; limb holds 90 (or 45) degrees, but drifts down before full 10 seconds; does not hit bed or other support<br>2 = Some effort against gravity; limb cannot get to or maintain (if cued) 90 (or 45) degrees, drifts down to bed, but has some effort against gravity<br>3 = No effort against gravity; limb falls<br>4 = No movement<br>9 = Amputation or joint fusion, explain:___
5b	Right motor arm:<br>Extend the arm: 90 degrees if sitting or 45 degrees if supine. Drift is scored if the arm falls before 10 seconds. The aphasic patient is encouraged using urgency in the voice and pantomime, but not noxious stimulation. The assessor can lift the patient's arm to the required position and encourage the patient to hold on. Assess the affected side only.	0 = No drift; limb holds 90 (or 45) degrees for full 10 seconds<br>1 = Drift; limb holds 90 (or 45) degrees, but drifts down before full 10 seconds; does not hit bed or other support<br>2 = Some effort against gravity; limb cannot get to or maintain (if cued) 90 (or 45) degrees, drifts down to bed, but has some effort against gravity<br>3 = No effort against gravity; limb falls<br>4 = No movement<br>9 = Amputation or joint fusion, explain:__
6a	Left motor leg:<br>Hold the leg at 30 degrees (always tested supine). Drift is	0 = No drift; leg holds 30-degree position for full 5 seconds

	Instructions	Score
	scored if the leg falls before 5 seconds. The aphasic patient is encouraged using urgency in the voice and pantomime, but not noxious stimulation. The assessor can lift the patient's leg to the required position and encourage the patient to hold on. Assess the affected side only.	1 = Drift; leg falls by the end of the 5-second period but does not hit bed<br>2 = Some effort against gravity; leg falls to bed by 5 seconds, but has some effort against gravity<br>3 = No effort against gravity; leg falls to bed immediately<br>4 = No movement<br>9 = Amputation or joint fusion, explain:__
6b	Right motor leg:<br>Hold the leg at 30 degrees (always tested supine). Drift is scored if the leg falls before 5 seconds. The aphasic patient is encouraged using urgency in the voice and pantomime, but not noxious stimulation. The assessor can lift the patient's leg to the required position and encourage the patient to hold on. Assess the affected side only.	0 = No drift; leg holds 30-degree position for full 5 seconds<br>1 = Drift; leg falls by the end of the 5-second period but does not hit bed<br>2 = Some effort against gravity; leg falls to bed by 5 seconds, but has some effort against gravity<br>3 = No effort against gravity; leg falls to bed immediately<br>4 = No movement<br>9 = Amputation or joint fusion, explain:__
7	Limb ataxia:<br>This item is aimed at finding evidence of a unilateral cerebellar lesion. Test with eyes open. In case of visual defect, ensure testing is done in intact visual field. The finger-nose-finger and heel-shin tests are performed on both sides, and ataxia is scored only if present out of proportion to weakness. Ataxia is absent in the patient who cannot understand or is paralyzed. In case of blindness, test by having the patient touch nose from extended arm position. Only in the case of amputation or joint fusion, the examiner will record the score as 9, and clearly write the explanation for this choice.	0 = Absent<br>1 = Present in one limb<br>2 = Present in two or more limbs
8	Sensory:<br>Examine with a pin. Sensation or grimace to pinprick when tested, or withdrawal from noxious stimulus in the obtunded or aphasic patient. Only sensory loss attributed to stroke is scored as abnormal. The examiner will test as many body areas: arms (not hands), legs, trunk, and face as needed to accurately check for hemisensory loss. A score of 2 "severe or total sensory loss" will only be given when a severe or total loss of sensation can be clearly demonstrated. Stuporous and aphasic patients will, therefore, probably score 1 or 0. The patient with brainstem stroke who has bilateral loss of sensation is scored 2. If the patient does not respond and is quadriplegic, score 2. Patients in a coma (item 1a = 3) are automatically given a 2 on this item.	0 = Normal; no sensory loss<br>1 = Mild-to-moderate sensory loss; patient feels pinprick is less sharp or is dull on the affected side; or there is a loss of superficial pain with pinprick, but patient is aware of being touched<br>2= Severe to total sensory loss; patient is not aware of being touched in the face, arm, and leg
9	Best language:<br>Naming and reading tests. The patient is asked to name the items on the attached naming sheet and to read from the attached list of sentences. Comprehension is judged from responses here, as well as to all the commands in the preceding general neurological exam. If visual loss	0 = No aphasia; normal<br>1 = Mild-to-moderate aphasia; some obvious loss of fluency or facility of comprehension, without significant limitation on ideas expressed or form of expression.<br>2 = Severe aphasia; all communication is

	Instructions	Score
	interferes with the tests, ask the patient to identify objects placed in the hand, repeat, and produce speech. The intubated patient will be asked to write. The patient in a coma (item 1a = 3) will automatically score 3 on this item. The examiner must choose a score for the patient with stupor or limited cooperation, but a score of 3 will be used only if the patient is mute and follows no one-step commands.	through fragmentary expression; great need for inference, questioning, and guessing by the listener. Range of information that can be exchanged is limited; listener carries burden of communication. Examiner cannot identify materials provided from patient response.<br>3 = Mute, global aphasia; no usable speech or auditory comprehension
10	Dysarthria:<br>Do not tell the patient why he or she is being tested. Ask the patient to read or repeat words from the attached list. If the patient has severe aphasia, the clarity of articulation of spontaneous speech can be rated. Only if the patient is intubated or has other physical barriers to producing speech, the examiner will record the score as 9, and clearly write the explanation for this choice.	0 = Normal<br>1 = Mild-to-moderate dysarthria; patient slurs at least some words and, at worst, can be understood with some difficulty<br>2 = Severe dysarthria; patient's speech is so slurred as to be unintelligible in the absence of or out of proportion to any dysphasia, or is mute/anarthric<br>9 = Intubated or other physical barriers, explain:__
11	Neglect:<br>If the patient has a severe visual loss preventing visual double simultaneous stimulation, and the cutaneous stimuli are normal, the score is normal. If the patient has aphasia but does appear to attend to both sides, the score is normal. Neglect is identified by examining the patient's ability to recognize simultaneous bilateral skin sensory and visual stimuli. Show the patient the standard picture and ask him/her to describe it. The doctor can encourage the patient to view carefully and identify features on the left and right. If the patient fails to recognize part of the image on one side, the score is abnormal. The doctor will then ask the patient to close his/her eyes and test response to pinprick in the arm or leg to examine bilateral skin sensation. The presence of sensory neglect on one side is taken as evidence of abnormality.	0 = No abnormality<br>1 = Visual, tactile, auditory, spatial, or personal inattention or extinction to bilateral simultaneous stimulation in one of the sensory modalities<br>2= Profound hemi-inattention or extinction to more than one modality; does not recognize own hand or orients to only one side of space

Appendix 4. EuroQoL Group 5-Dimension Questionnaire

Under each heading, please tick the one box that best describes your health today	
Mobility	☐1 I have no problems in walking about<br>☐2 I have some problems in walking about<br>☐3 I am confined to bed
Self-care	☐1 I have no problems with self-care<br>☐2 I have some problems washing, bathing, or dressing myself<br>☐3 I am unable to wash, bath, or dress myself
Usual activities<br>(e.g., work, study, housework, family, or leisure activities)	☐1 I have no problems with performing my usual activities<br>☐2 I have some problems with performing my usual activities<br>☐3 I am unable to perform my usual activities
Pain/Discomfort	☐1 I have no pain or discomfort<br>☐2 I have moderate pain or discomfort<br>☐3 I have extreme pain or discomfort
Anxiety/Depression	☐1 I am not anxious or depressed<br>☐2 I am moderately anxious or depressed<br>☐3 I am extremely anxious or depressed

To help you know how good or bad your health is today; we draw a scale (kind of like a temperature meter). On this scale, 100 means the best health you can imagine, and 0 means the worst health you can imagine.

Please make a mark on the scale on the right to indicate how your health is today. Please draw a line from one of the boxes below to the point on the scale that can best represent your health today.

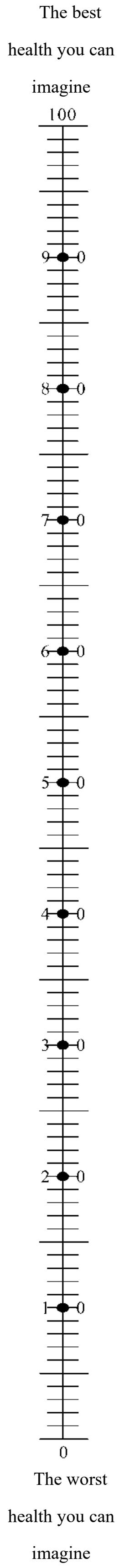

YOUR HEALTH TODAY

The questionnaire is completed by:

☐1 Patient

☐2 Patient with the assistance of a third person

☐3 Proxy (patient's family member)

Appendix 5. Barthel Index

Item	Scoring criteria
Feeding	☐0 Dependent<br>☐5 Needs some help<br>☐10 Independent
Bathing	☐0 Dependent<br>☐5 Independent
Grooming (face, hair, teeth, shaving)	☐0 Needs help<br>☐5 Independent
Dressing (buttons, zips, laces)	☐0 Dependent<br>☐5 Needs some help<br>☐10 Independent
Bowels	☐0 Incontinent or needs to be given enemas<br>☐5 Occasional accident<br>☐10 Continent
Bladder	☐0 Incontinent or catheterized and unable to manage alone<br>☐5 Occasional accident<br>☐10 Continent
Toilet use (on and off, dressing, wiping)	☐0 Dependent<br>☐5 Needs some help<br>☐10 Independent
Transfers (bed to chair and back)	☐0 Unable, no sitting balance<br>☐5 Major help (2 people, physical), can sit<br>☐10 Minor help (1 person, verbal or physical)<br>☐15 Independent
Mobility (on level surfaces)	☐0 Immobile or < 45 meters<br>☐5 Wheelchair independent, including corners, > 45 meters<br>☐10 Walks with help of 1 person (verbal or physical) > 45 meters<br>☐15 Independent (but may use any aid) > 45 meters
Stairs	☐0 Unable<br>☐5 Needs help (physical, verbal, carrying aid)<br>☐10 Independent
