# Supplementary material for: Efficacy of Panax notoginseng saponins on functional outcome in obese patients with acute ischemic stroke
Source: J Ginseng Res. 2026 Feb 6;50(3):100991. doi: 10.1016/j.jgr.2026.100991 (PMC13149892; doi:10.1016/j.jgr.2026.100991)
Supplement: Multimedia component 2 [file mmc2.pdf]

# Supplementary Material 2

# Network Pharmacology Analysis of Panax Notoginseng Saponins in Ischemic Stroke with Obesity

## Chemical Structure and Target Prediction of PNS Components

The bioactive components of Panax notoginseng saponins (PNS)—ginsenoside Rb1, ginsenoside Rd, ginsenoside Rg1, ginsenoside Re, and notoginsenoside R1—were retrieved in structure from the PubChem database (https://pubchem.ncbi.nlm.nih.gov/). These structures were subsequently submitted to the PharmMapper database (https://www.lilab-ecust.cn/pharmmapper/) for target prediction. PharmMapper employs a reverse pharmacophore matching approach to identify potential protein targets based on molecular structure. High-ranking targets were selected according to normalized fit scores for further analysis.

## Collection of Disease-Related Targets

Targets associated with ischemic stroke (IS) and obesity were collected from public databases including OMIM (https://www.omim.org/), GeneCards (https://www.genecards.org/), and DrugBank (https://go.drugbank.com/). The keywords "ischemic stroke" and "obesity" were used to query relevant gene targets. Duplicate entries were removed to generate unique gene sets for each condition.

## Screening of Common Targets

Common targets among PNS, IS, and obesity were identified by intersecting the predicted targets of PNS with the disease-associated targets using a Venn diagram tool (http://www.bioinformatics.com.cn).

## Compound-Target Interaction Network

A compound-target interaction network was constructed using Cytoscape (version 3.8.2). Node connectivity was evaluated through topological analysis, with the degree value used to identify hub targets. This helped pinpoint the most influential targets within the network.

## Protein-Protein Interaction (PPI) Network Analysis

Common targets were imported into the STRING database (https://string-db.org/) to construct a PPI network under a confidence score threshold ≥ 0.4. The resulting network was visualized and analyzed in Cytoscape to identify highly connected hub proteins.

## Functional and Pathway Enrichment Analysis

Gene Ontology (GO) and Kyoto Encyclopedia of Genes and Genomes (KEGG) pathway enrichment analyses were performed using Metascape (https://metascape.org/). Significantly enriched terms and pathways ($P < 0.05$) were visualized to elucidate biological functions, molecular mechanisms, and key signaling pathways.

## Results

Target prediction via PharmMapper yielded 211 potential protein targets for PNS. A total of 3,745 IS-related targets and 2,825 obesity-related targets were compiled from disease databases. Among these, 1,752 targets overlapped between IS and obesity. Venn analysis identified 83 common targets shared among PNS, IS, and obesity (**Figure a**). The compound-target network is illustrated in **Figure b**. The top ten potential targets included ALB, IGF1, PPARG, ESR1, MMP2, BMP2, DHFR, SERPINA1, PTPN, and DPP4 (**Figure c**). KEGG analysis revealed 34 significantly enriched pathways ($P < 0.05$), with the top five being Proteoglycans in cancer, HIF-1 signaling pathway, Phenylalanine metabolism, Insulin resistance, and the Estrogen signaling pathway (**Figure d**). GO analysis identified 19 cellular components, 39 molecular functions, and 383 biological processes ($P < 0.05$, Figure 2e). These results suggest that PNS may ameliorate ischemic stroke in obese patients through mechanisms involving oxidative stress, inflammatory response, insulin resistance, microcirculation dysfunction, and neuronal damage.

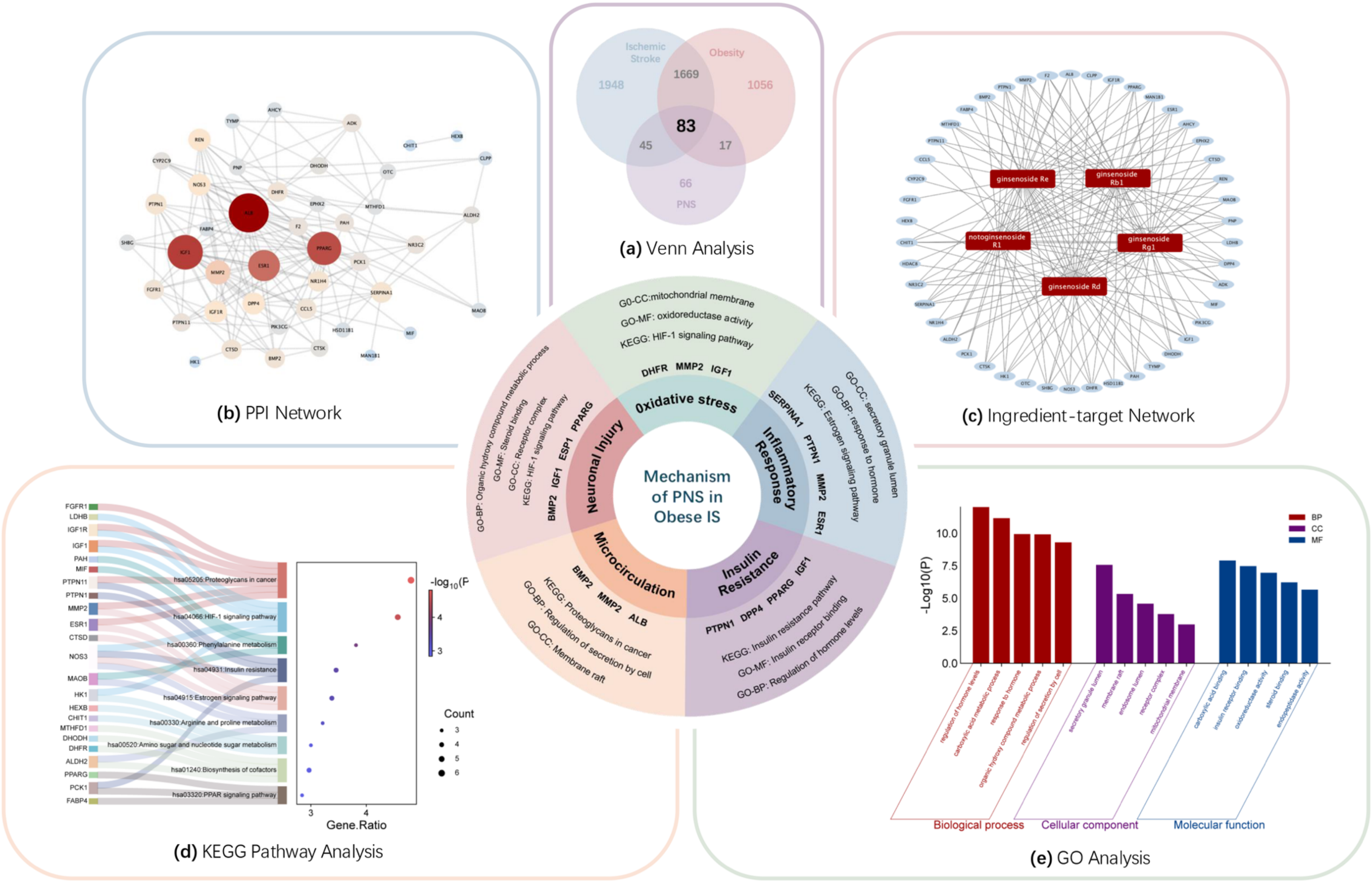

(a) Venn Analysis
Ischemic Stroke
Obesity
PNS
1948
1669
1056
83
45
17
66
(b) PPI Network
(c) Ingredient-target Network
ginsenoside Re
ginsenoside Rb1
notoginsenoside R1
ginsenoside Rg1
ginsenoside Rd
Mechanism of PNS in Obese IS
Oxidative stress
DHFR MMP2 IGF1
G0-CC:mitochondrial membrane
GO-MF: oxidoreductase activity
KEGG: HIF-1 signaling pathway
Inflammatory Response
SERPINA1 PTPN1 MMP2 ESR1
GO-CC: secretory granule lumen
GO-BP: response to hormone
KEGG: Estrogen signaling pathway
Insulin Resistance
PTPN1 DPP4 PPARG IGF1
KEGG: Insulin resistance pathway
GO-MF: Insulin receptor binding
GO-BP: Regulation of hormone levels
Microcirculation
BMP2 MMP2 ALB
KEGG: Proteoglycans in cancer
GO-BP: Regulation of secretion by cell
GO-CC: Membrane raft
Neuronal Injury
BMP2 IGF1 ESP1 PPARG
GO-BP: Organic hydroxy compound metabolic process
GO-MF: Steroid binding
GO-CC: Receptor complex
KEGG: HIF-1 signaling pathway
(d) KEGG Pathway Analysis
hsa05205:Proteoglycans in cancer
hsa04066:HIF-1 signaling pathway
hsa00360:Phenylalanine metabolism
hsa04931:Insulin resistance
hsa04915:Estrogen signaling pathway
hsa00330:Arginine and proline metabolism
hsa00520:Amino sugar and nucleotide sugar metabolism
hsa01240:Biosynthesis of cofactors
hsa03320:PPAR signaling pathway
Gene.Ratio
Count
(e) GO Analysis
-Log10(P)
BP
CC
MF
Biological process
Cellular component
Molecular function

**Figure. (a)**Venn diagram analysis between the PNS-predicted targets and the shared disease targets (IS and obesity). **(b)** The protein-protein interaction (PPI) network of shared disease targets. **(c)** The ingredient-target network between PNS, IS and obesity. **(d)** Enrichment analysis of the target Kyoto Encyclopedia of Genes and Genomes **(**KEGG) pathway. **(e)** Histogram of Gene Ontology **(**GO) analysis of targets.

66
