# Supplementary material for: Efficacy of Panax notoginseng saponins on functional outcome in obese patients with acute ischemic stroke
Source: J Ginseng Res. 2026 Feb 6;50(3):100991. doi: 10.1016/j.jgr.2026.100991 (PMC13149892; doi:10.1016/j.jgr.2026.100991)
Supplement: Multimedia component 3 [file mmc3.pdf]

김 민준

325894

데이터과학39

Document Details

Submission ID

trn:oid:::3618:125169108

Submission Date

Dec 26, 2025, 12:22 PM GMT+9

Download Date

Dec 26, 2025, 12:26 PM GMT+9

File Name

176671929428698.docx

File Size

82.2 KB

14 Pages

2,698 Words

16,175 Characters

17% Overall Similarity

The combined total of all matches, including overlapping sources, for each database.

Filtered from the Report

- Bibliography
- Quoted Text

Match Groups

48 Not Cited or Quoted 17%
Matches with neither in-text citation nor quotation marks

0 Missing Quotations 0%
Matches that are still very similar to source material

0 Missing Citation 0%
Matches that have quotation marks, but no in-text citation

0 Cited and Quoted 0%
Matches with in-text citation present, but no quotation marks

Top Sources

15% Internet sources
10% Publications
11% Submitted works (Student Papers)

Integrity Flags

0 Integrity Flags for Review

Our system's algorithms look deeply at a document for any inconsistencies that would set it apart from a normal submission. If we notice something strange, we flag it for you to review.

A Flag is not necessarily an indicator of a problem. However, we'd recommend you focus your attention there for further review.

Match Groups

- **48** Not Cited or Quoted 17%
  Matches with neither in-text citation nor quotation marks
- **0** Missing Quotations 0%
  Matches that are still very similar to source material
- **0** Missing Citation 0%
  Matches that have quotation marks, but no in-text citation
- **0** Cited and Quoted 0%
  Matches with in-text citation present, but no quotation marks

Top Sources

15% Internet sources
10% Publications
11% Submitted works (Student Papers)

Top Sources

The sources with the highest number of matches within the submission. Overlapping sources will not be displayed.

#	Type	Source	%
1	Internet	www.omicsdi.org	2%
2	Internet	pmc.ncbi.nlm.nih.gov	2%
3	Internet	www.frontiersin.org	1%
4	Internet	www.mdpi.com	<1%
5	Internet	bmcwomenshealth.biomedcentral.com	<1%
6	Internet	www.mdedge9-ma1.mdedge.com	<1%
7	Internet	www.researchgate.net	<1%
8	Publication	Edgar Antonio Ramos Gutierrez, Mario Ulises Pérez Zepeda, Natalia Sánchez Garri...	<1%
9	Student papers	The University of Texas at Arlington on 2025-11-27	<1%
10	Internet	www.sciencerepository.org	<1%

11	Internet	
link.springer.com		<1%
12	Internet	
medtechnews.uk		<1%
13	Internet	
escholarship.org		<1%
14	Publication	
Hsu, Tai-Yi, Yi-Ming Weng, Yu-Hui Chiu, Wen-Cheng Li, Pang-Yen Chen, Shih-Hao ...		<1%
15	Internet	
www.spandidos-publications.com		<1%
16	Student papers	
University of Sheffield on 2025-09-17		<1%
17	Publication	
Wen Wei, Xie Xin, Bing Shao, Fang-Fang Zeng, Edgar J Love, Bin-You Wang. "The r...		<1%
18	Internet	
www.gospel10.com		<1%
19	Internet	
zaguan.unizar.es		<1%
20	Internet	
jnfh.mums.ac.ir		<1%
21	Internet	
www.eurosurveillance.org		<1%
22	Internet	
www.science.gov		<1%
23	Internet	
pesquisa.bvsalud.org		<1%
24	Publication	
K Kimura. "Atrial fibrillation as a predictive factor for severe stroke and early dea...		<1%

25 Publication

Ying Wang, Juan Yang, Yuzhen Liu, Ao Yang, Yuqing Deng, Chang Xu, Shilin Zhong... <1%

26 Internet

www.researchsquare.com <1%

27 Publication

"Full Issue PDF", JACC: Asia, 2025 <1%

28 Student papers

Flinders University on 2024-04-12 <1%

29 Student papers

University of North Texas on 2024-09-20 <1%

30 Publication

Xin Zhang, Xiaoyong He, Fan Mao, Run Zhang, Xiaoqing You, Jianhong Li. "Relatio... <1%

31 Publication

Jian-Qiang Tan, Li-Mei Gu, Yan-Ling Zhou, Cheng-Yu Wang, Xiao-Feng Lan, Wei Zh... <1%

32 Student papers

University of Bath on 2012-12-06 <1%

33 Internet

www.ncbi.nlm.nih.gov <1%

Efficacy of Panax Notoginseng Saponins on Functional Outcomes in Patients with Obesity and Acute Ischemic Stroke

Abstract

Background

Obesity exacerbates acute ischemic stroke (AIS) outcomes through metabolic dysfunction and chronic inflammation. Although Panax notoginseng saponins (PNS) have demonstrated efficacy in the treatment of AIS in the large-scale PANDA trial (N = 3072; ChiCTR1800016363), their benefits in patients with obesity specifically, remain unclear. This study aimed to evaluate the effect of PNS on functional outcomes in patients with obesity.

Methods

This analysis utilized individual patient data from the PANDA trial. Participants were stratified by body mass index (BMI), waist circumference (WC), **and a combination of both metrics**. The primary outcome was functional independence, defined as a modified Rankin Scale (mRS) score of 0–2 at 90 days. Adjusted odds ratios (aORs) were calculated using multivariable logistic regression.

Results

Among 2779 patients (mean age 60.7 ± 9.3 years), 58.3% were classified as overweight or obese by BMI, and 65% met criteria for abdominal obesity based on WC. PNS significantly improved rates of functional independence at 90 days across overweight (aOR = 2.05; 95% CI: 1.39–3.06), obesity (aOR = 2.18; 95% CI: 1.11–4.41), and abdominal obesity (aOR = 2.37; 95% CI: 1.70–3.28) subgroups. Consistent benefits were observed in patients with abdominal obesity irrespective of BMI category: lower BMI (aOR = 2.45; 95% CI: 1.34–4.61) and higher BMI (aOR = 2.40; 95% CI: 1.64–3.54).

Conclusions

These results indicate that PNS may improve 90-day functional outcomes in patients with AIS and obesity, including those with abdominal obesity, warranting further prospective validation.

Keywords Acute Ischemic Stroke, Panax Notoginseng Saponins, Abdominal Obesity, Obesity

1. Introduction

Acute ischemic stroke (AIS) is a leading cause of mortality and disability worldwide[1]. Concurrently, obesity has become a critical public health challenge, affecting more than two billion people globally[2]. Evidence indicates that obesity increases the risk of stroke and adversely affects functional recovery post-stroke[3–5]. Particularly, abdominal obesity, characterized by excessive visceral adipose tissue, has been a well-established independent risk factor for stroke[6]. Recent studies suggest that abdominal obesity is associated with poor neurological outcomes after AIS[7]. **This impaired recovery is often attributed to chronic inflammation, endothelial dysfunction, and metabolic disturbances such as insulin resistance, which are hallmarks of obesity that can exacerbate secondary brain injury and hinder neurorepair**[8–12]. Given the escalating prevalence of obesity and its compounding effect on stroke-related disability, identifying strategies to improve functional recovery in this population represents an urgent clinical priority.

Panax notoginseng (Burk.) F.H. Chen, which is referred to as Sanqi in China, is a perennial herb belonging to the Araliaceae family. It has been used in traditional medicine for more than 400 years. Panax notoginseng saponins (PNS), the main bioactive constituents of this herb, are widely employed for the treatment of cardiovascular and cerebrovascular diseases. PNS includes several saponins such as ginsenosides Rg1, Rd, Rb1, Re, and notoginsenoside R1, which mediate therapeutic effects through anti-inflammatory[13], antioxidant[14,15], pro-neurogenic[16], and pro-angiogenic[17] mechanisms following cerebral ischemia. These multifaceted mechanisms have established PNS as a compound of significant interest in pharmacological research worldwide. More recently, PNS has garnered attention for its potential metabolic benefits, including improved insulin sensitivity and modulation of obesity-related pathological processes,

suggesting its potential applicability in patients with obesity and AIS[18–20]. The PANDA trial (N=3072), which is the largest multicenter randomized controlled trial of PNS in patients with AIS to date, demonstrated improved functional outcomes at 90 days without increasing adverse events[21]. **Although the main PANDA trial established the overall efficacy of PNS in patients with AIS, it remains unclear whether this benefit extends uniformly across the growing population of patients with obesity and stroke, who are at increased risk of suboptimal recovery. Therefore, we performed a post-hoc analysis to investigate the efficacy of PNS in patients with obesity and AIS, an inquiry not addressed in the primary trial report, with significant implications for personalized post-stroke management.**

2. Material and methods

2.1. Study Design

This analysis utilized individual data from the original PANDA trial, which evaluated the efficacy of PNS in ischemic stroke. The PANDA trial was a randomized, double-blind, placebo-controlled study involving 3,072 participants (Registration No.: ChiCTR1800016363; chictr.org.cn). Randomization was performed using a centralized randomization system to ensure allocation concealment. The study design and primary outcomes have been previously reported[21,22]. Further protocol details are provided in **Supplementary Material 1**. The study adhered to strict ethical standards, and all patients provided written informed consent before participating in any study-related procedures. Ethical approval was granted by the Ethics Committee of Xuanwu Hospital, Capital Medical University (Approval No.: LYS[2018]005). The flow chart of this study was presented in **Figure 1**.

2.2. Population and Treatment

Eligible participants were aged between 18 and 75 years, diagnosed with AIS, had a pre-stroke modified Rankin Scale (mRS) score of ≤1, and a National Institutes of Health Stroke Scale (NIHSS) score between 4 and 15 at the time of randomization. Patients were randomly assigned to either the PNS group or the placebo group.

2.3. Anthropometric Measures

Body Mass Index (BMI) and Waist Circumference (WC) were used as the standard of overall obesity and abdominal obesity, respectively. Data on height, weight, and WC were collected after randomization. BMI was calculated as weight in kilograms divided by the square of height in meters (kg/m^2). Criteria for BMI and WC categories were based on the Chinese Guidelines for the

Clinical Management of Obesity (2024 edition). **Chinese BMI criteria were applied in this study because the study population was exclusively Chinese, and Asian populations exhibit a higher percentage of body fat and increased risk of obesity-related comorbidities at a given BMI compared with other populations[23]; therefore, population-specific criteria are more accurate for risk stratification.** Patients were stratified as follows: (1) According to BMI: underweight (BMI < 18.5 kg/m²), normal weight (18.5 ≤ BMI < 24 kg/m²), overweight (24 ≤ BMI < 28 kg/m²), and obese (BMI ≥ 28 kg/m²); (2) According to WC: normal WC (men < 85 cm, women < 80 cm) and abdominal obesity (men ≥ 85 cm, women ≥ 80 cm); (3) Combined stratification: lower BMI (BMI < 24 kg/m²) with normal WC, higher BMI (BMI ≥ 24 kg/m²) with normal WC, lower BMI (BMI < 24 kg/m²) with abdominal obesity, and higher BMI (BMI ⩾ 24 kg/m²) with abdominal obesity.

2.4. Outcomes

The primary efficacy outcome was the rate of functional independence, defined as mRS ⩽2, at 3 months. The secondary efficacy outcomes were as follows: (1) the rate of functional independence at 12 months; and (2) the proportion of no or minimal disability, defined as mRS⩽1, at 3 and 12 months.

2.5. Statistical Analysis

All randomized participants who received at least one dose of the study drug (PNS or placebo) and had at least one assessment of the primary efficacy outcome, were included in the intent-to-treat (ITT) population. All analyses were performed on this population.

Baseline characteristics were analyzed using χ^2 tests for categorical data and one-way analysis of variance for continuous variables, and presented as frequencies (percentages) and mean ± SD,

respectively. After adjustment for sex, age, diastolic blood pressure, history of ischemic stroke, vasospastic angina, hyperlipidemia, hypertension, diabetes, and NIHSS score at randomization, multivariable logistic regression was used to assess the associations between BMI/WC categories (individually and combined) and functional outcomes, reporting adjusted odds ratios (ORs) with 95% confidence intervals (CIs). Data analysis was performed using the R software (v4.3.2). Statistical significance for all two-tailed tests was established at a P-value of <0.05.

3. Results

3.1. Baseline characteristics

Among the 2,779 patients with available BMI and WC data (927 [33.4%] women; mean age, 60.7 ± 9.3 years), 1,557 (56%) presented with functional independence (an mRS score of 0–2) at randomization. Obesity was highly prevalent: 1,620 patients (58.3%) were overweight or obese according to BMI criteria, and 1,807 (65%) met the criteria for abdominal obesity based on WC. **Notably, among the 1,159 patients with a normal BMI, 520 (44.9%) had abdominal obesity.** Only 536 patients (19.3%) had both, a normal BMI and a normal WC. Detailed baseline characteristics stratified by BMI, WC, and their combination are provided in **Tables S1**, **S2**, and **S3**.

3.2. Efficacy of PNS in Acute Ischemic Stroke with Comorbid Obesity

The detailed primary outcomes are shown in **Table 1** and **Figure 2**, while the secondary outcomes are provided in **Table S4**.

3.2.1. Efficacy Outcomes Stratified by BMI

PNS was associated with a higher proportion of functional independence, in patients with a higher BMI. Among overweight patients, the rate of functional independence was 91.0% in the PNS

group compared to 84.0% in the placebo group (adjusted odds ratio [aOR] = 2.05 [95% CI, 1.39–3.06]). A similar benefit was observed in patients with obesity (90.9% vs. 82.0%; aOR = 2.18 [95% CI, 1.11–4.41]). Additionally, in the overweight cohort, the PNS group had a significantly higher proportion of patients with no or minimal disability at 12 months (86.5% vs. 81.9%; aOR = 1.45 [95% CI, 1.04–2.05]).

3.2.2. Efficacy Outcomes Stratified by WC

Additionally, PNS was associated with significant improvements in patients with abdominal obesity. The functional independence rate was 90.3% in the PNS group versus 83.2% in the placebo group (aOR = 2.37 [95% CI, 1.73-3.28]). Furthermore, PNS treatment increased the proportion of patients with no or minimal disability at 3 months (74.3% vs. 70.1%; aOR = 1.34 [95% CI, 1.06-1.70]) and 12 months (85.4% vs. 82.0%; aOR = 1.40 [95% CI, 1.07–1.84]) in this group.

3.2.3. Efficacy Outcomes Stratified by BMI and WC

The effect of PNS in improving 3-month functional independence were significant in the abdominal obesity subgroups, regardless of the BMI status (lower BMI: aOR = 2.45 [95% CI, 1.34–4.61]; higher BMI: aOR = 2.40 [95% CI, 1.64–3.54]). Conversely, no significant benefit was observed in patients without abdominal obesity across both BMI strata (lower BMI: aOR = 1.57 [95% CI, 0.95–2.60]; higher BMI: aOR = 1.73 [95% CI, 0.79–3.89]).

4. Discussion

The analysis in the present study yielded two principal findings: first, PNS significantly improved the rate of functional independence at 3 months in patients with overall obesity and those with abdominal obesity; second, the treatment effect was numerically higher in the abdominal obesity subgroup, suggesting a potential trend that warrants further investigation. These findings are further supported by the secondary outcome analysis focusing on longer-term recovery. PNS was associated with a higher proportion of individuals achieving no or minimal disability at 12 months, with an increase of 4.6% in the overweight subgroup and 3.4% in the abdominal obesity subgroup. These consistent benefits on long-term functional outcomes underscore the durable therapeutic effect of PNS in this clinically challenging population.

Abdominal obesity is increasingly recognized as an independent factor that compromises therapeutic efficacy across multiple treatment modalities, posing considerable challenges to optimal stroke recovery[6,24,25]. Therefore, patients in the present study were further stratified using WC to capture abdominal obesity more accurately. Notably, PNS remained effective in this analysis, demonstrating an absolute improvement of 7.1% in the rate of functional independence among patients with abdominal obesity. These concordant findings across different anthropometric measures strengthen the evidence for PNS efficacy in this high-risk population, that often demonstrates suboptimal responses to conventional therapies.

When viewed in the context of existing research, our findings hold significant importance. Previous studies have consistently demonstrated that patients with obesity experiencing AIS respond inadequately to conventional treatments. A consecutive cohort study of 304 patients receiving intravenous thrombolysis therapy showed that obesity led to a lower rate of favorable outcomes following therapy by 17.2%[26]. Furthermore, a study using abdominal computed

tomography to accurately quantify visceral adipose tissue (VAT) revealed that among patients with AIS receiving guideline-recommended treatment, those in the highest VAT group had a 14.5% lower probability of achieving a favorable outcome compared with the lowest VAT group[25]. Another study focusing on abdominal fat distribution further confirmed that abdominal obesity reduces the efficacy of endovascular therapy, leading to a decrease in the proportion of patients achieving favorable functional outcomes by 8.5% at 90 days, and 12.2% at one year[24]. In contrast to these reports of attenuated treatment responses in populations with obesity, our exploratory analysis found that PNS was associated with a significant benefit in the abdominal obesity subgroup. This marked divergence from the established pattern suggests that PNS may offer a novel therapeutic strategy.

The therapeutic benefit of PNS in patients with obesity and AIS is attributed to their multi-target pharmacological actions that counter obesity-aggravated pathophysiological processes. Notably, PNS contains ginsenosides Rg1, Rd, Rb1, Re, and notoginsenoside R1 which collectively exhibit anti-inflammatory, antioxidant, anti-apoptotic, and blood–brain barrier restorative effects[16,27,28]. Importantly, these compounds mitigate the central mechanisms of stroke injury exacerbated by obesity, such as oxidative stress and inflammation, by reducing ROS accumulation, upregulating hypoxia-inducible factor-1α[19,29,30], and modulating cytokine expression through suppression of IL)-1β and TNF-α) alongside elevation of IL-10[13]. Furthermore, PNS promotes vascular repair by enhancing angiogenesis and micro-perfusion via AMPK- and eNOS)dependent pathways[31]. Particularly, ginsenoside Rb1 demonstrates potent antioxidant activity through binding to estrogen receptor-β, thereby activating protective signaling that restores eNOS and superoxide dismutase expression[15]. Moreover, abdominal obesity is closely linked to insulin resistance, a condition that exacerbates neuroinflammation, endothelial dysfunction, and impaired

recovery after stroke[9,32]. The multi-target pharmacological profile of PNS is further complemented by its potential to confer metabolic benefits. These benefits, primarily mediated through the modulation of neuroprotective, anti-oxidant, anti-inflammatory, micro-circulatory, and insulin resistance pathways, are consistent with predictions from previous network pharmacology analysis by the authors (**Supplementary Material 2**). Additionally, preliminary reports suggest that PNS improves insulin sensitivity and regulates metabolic homeostasis[16–18]; however, clinical validation in stroke patients remains necessary to substantiate these mechanistic insights. In summary, the synergistic modulation of neuroinflammatory, oxidative, metabolic, and vascular repair pathways emphasize the efficacy of PNS in obese stroke patients. This multi-mechanistic action highlights the need for further investigation of PNS as a potential therapy for the population that often responds poorly to conventional treatments.

The strengths of the present study include the use of data from the PANDA trial, the largest randomized trial of PNS in AIS, which ensures high-quality and standardized data collection. To the best of our knowledge, this is the largest study to provide evidence supporting the efficacy of PNS in patients with obesity and AIS. The combined use of BMI and WC enhances the robustness of our conclusions against bias from any single anthropometric measurement.

Several limitations of the present study should be considered. First, this was a post-hoc analysis, and the investigation of obesity subgroups was not pre-specified in the original PANDA trial protocol. Therefore, the findings should be interpreted as exploratory and hypothesis-generating rather than confirmatory of a causal relationship. Second, the study relied on data from a Chinese cohort and excluded individuals with severe obesity (BMI > 35 kg/m²), which may have affected the generalizability of these results. Third, the study lacked serial data on metabolic parameters such as BMI, WC, and lipid profiles at the 90-day follow-up. Therefore, we could not

directly investigate whether the observed clinical benefits were mediated through the modulation of adiposity-related metabolic pathways. This important mechanistic question should be addressed in future prospective studies. Moreover, the BMI categorization in this study was based on criteria specific to the Chinese population, which may limit direct comparability with studies using international BMI standards. Finally, formal tests for interaction between treatment group and obesity status did not yield statistically significant results in either the BMI or abdominal obesity subgroups, suggesting that the beneficial effect of PNS was generally consistent across these patient categories. Nonetheless, the adjusted odds ratios indicated a trend toward a more pronounced treatment effect in patients with abdominal obesity. The absence of a significant interaction may be attributed to reduced statistical power resulting from subgroup stratification. Therefore, our observation of a potentially enhanced benefit in this population should be interpreted with caution and warrants further validation in future, adequately powered prospective studies.

turnitin Page 17 of 19 - Integrity Submission Submission ID trn:oid:::3618:125169108

5. Conclusion

PNS is associated with improved functional outcomes at 3 months in patients with obesity and AIS, with a potentially enhanced benefit observed in those with abdominal obesity. These exploratory findings suggest that investigating PNS as a potential adjunct strategy for this population is warranted, and requires further validation in prospective studies

234
