# Supplementary material for: Efficacy of Panax notoginseng saponins on functional outcome in obese patients with acute ischemic stroke
Source: J Ginseng Res. 2026 Feb 6;50(3):100991. doi: 10.1016/j.jgr.2026.100991 (PMC13149892; doi:10.1016/j.jgr.2026.100991)
Supplement: Multimedia component 4 [file mmc4.docx]

**Table S1 Baseline Characteristics Stratified by BMI**

|  | **Underweight** | | | **Normal weight** | | | **Overweight** | | | **Obesity** | | |
| --- | --- | --- | --- | --- | --- | --- | --- | --- | --- | --- | --- | --- |
|  | **(n=54)** | | | **(n=1105)** | | | **(n=1234)** | | | **(n=386)** | | |
|  | **PNS** | **Placebo** | ***P* value** | **PNS** | **Placebo** | ***P* value** | **PNS** | **Placebo** | ***P* value** | **PNS** | **Placebo** | ***P* value** |
|  | **(n=27)** | **(n=27)** |  | **(n=547)** | **(n=558)** |  | **(n=625)** | **(n=609)** |  | **(n=189)** | **(n=197)** |  |
| **Male Sex, n (%)** | 16(59.3) | 20(74.1) | 0.386 | 373(68.2) | 368(65.9) | 0.466 | 458(73.3) | 379(62.2) | ＜0.001^***^ | 114(60.3) | 124(62.9) | 0.670 |
| **Age, mean (SD), years** | 66.48(7.15) | 64.48(8.33) | 0.348 | 62.13(9.11) | 61.63(8.71) | 0.345 | 60.05(9.16) | 60.84(9.31) | 0.136 | 57.38(10.08) | 58.22(10.05) | 0.411 |
| **Heart rate, mean (SD)** | 73.89(13.90) | 74.41(7.76) | 0.866 | 75.47(10.04) | 76.20(10.99) | 0.253 | 75.46(9.83) | 75.12(9.74) | 0.539 | 75.81(11.49) | 74.82(9.93) | 0.361 |
| **SBP, mean (SD), mmHg** | 138.37(20.44) | 139.70(17.82) | 0.799 | 144.12(19.34) | 142.92(18.80) | 0.295 | 145.02(19.07) | 145.32(16.90) | 0.767 | 148.65(19.09) | 147.86(17.87) | 0.676 |
| **DBP, mean (SD), mmHg** | 77.15(8.13) | 82.63(10.68) | 0.039^*^ | 84.24(12.29) | 84.01(12.18) | 0.756 | 86.86(12.19) | 85.52(11.61) | 0.049^*^ | 89.23(12.46) | 88.35(13.14) | 0.499 |
| **mRS ≤2 at randomization, n(%)** | 15(55.6) | 17(63.0) | 0.782 | 307(56.1) | 307(55.0) | 0.757 | 363(58.1) | 328(53.9) | 0.151 | 113(59.8) | 107(54.3) | 0.326 |
| **NIHSS at randomization, mean (SD)** | 6.26(2.92) | 5.63(2.06) | 0.364 | 5.97(2.37) | 6.05(2.49) | 0.562 | 5.88(2.39) | 5.75(2.25) | 0.309 | 5.79(2.13) | 5.90(2.32) | 0.644 |
| **IS, n (%)** | 7(25.9) | 1(3.7) | 0.055 | 102(18.6) | 81(14.5) | 0.077 | 110(17.6) | 109(17.9) | 0.950 | 35(18.5) | 37(18.8) | 1.000 |
| **TIA, n (%)** | 0(0.0) | 0(0.0) | — | 6(1.1) | 3(0.5) | 0.484 | 5(0.8) | 2(0.3) | 0.469 | 1(0.5) | 0(0.0) | 0.983 |
| **VSA, n (%)** | 1(3.7) | 0(0.0) | 1.000 | 17(3.1) | 11(2.0) | 0.312 | 10(1.6) | 13(2.1) | 0.629 | 7(3.7) | 1(0.5) | 0.065 |
| **Hyperlipidemia, n (%)** | 1(3.7) | 1(3.7) | 1.000 | 35(6.4) | 15(2.7) | 0.005 | 30(4.8) | 40(6.6) | 0.223 | 15(7.9) | 10(5.1) | 0.350 |
| **Hypertension, n (%)** | 13(48.1) | 8(29.6) | 0.264 | 266(48.6) | 277(49.6) | 0.782 | 376(60.2) | 377(61.9) | 0.569 | 134(70.9) | 132(67.0) | 0.474 |
| **Diabetes, n (%)** | 7(25.9) | 3(11.1) | 0.293 | 126(23.0) | 125(22.4) | 0.858 | 161(25.8) | 171(28.1) | 0.393 | 55(29.1) | 49(24.9) | 0.412 |
| **Current smoking, n (%)** | 8(29.6) | 5(18.5) | 0.524 | 155(28.3) | 153(27.4) | 0.785 | 175(28.0) | 149(24.5) | 0.178 | 47(24.9) | 43(21.8) | 0.558 |
| **Current drinking, n (%)** | 3(11.1) | 2(7.4) | 1.000 | 78(14.3) | 76(13.6) | 0.826 | 79(12.6) | 83(13.6) | 0.667 | 23(12.2) | 34(17.3) | 0.206 |
| **pre-mRS =0, n (%)** | 17(63.0) | 18(66.7) | 1.000 | 390(71.3) | 382(68.5) | 0.336 | 463(74.1) | 449(73.7) | 0.939 | 140(74.1) | 152(77.2) | 0.557 |

**Abbreviations:** BMI, body mass index; DBP, diastolic blood pressure; IS, ischaemic stroke; mRS, modified Rankin scale; NIHSS, National Institutes of Health Stroke Scale; PNS, *Panax Notoginseng* Saponins; SBP, systolic blood pressure; TIA, transient ischemic attack; VSA, vasospastic angina; WC, waist circumference.

**P* value＜0.05；***P* value＜0.01; ****P* value＜0.001.
