# Supplementary material for: Efficacy of Panax notoginseng saponins on functional outcome in obese patients with acute ischemic stroke
Source: J Ginseng Res. 2026 Feb 6;50(3):100991. doi: 10.1016/j.jgr.2026.100991 (PMC13149892; doi:10.1016/j.jgr.2026.100991)
Supplement: Multimedia component 5 [file mmc5.docx]

**Table S2 Baseline Characteristics Stratified by WC**

|  | **Normal WC** | | | **Abdominal Obesity** | | |
| --- | --- | --- | --- | --- | --- | --- |
|  | **(n=972)** | | | **(n=1807)** | | |
|  | **PNS** | **Placebo** | ***P* value** | **PNS** | **Placebo** | ***P* value** |
|  | **(n=484)** | **(n=488)** |  | **(n=904)** | **(n=903)** |  |
| **Male Sex, n (%)** | 350 (71.7) | 360 (74.4) | 0.389 | 541 (59.9) | 601 (66.5) | 0.004^**^ |
| **Age, mean (SD), years** | 62.26 (8.90) | 61.51 (9.65) | 0.207 | 60.09 (9.33) | 60.16 (9.23) | 0.871 |
| **Heart rate, mean (SD)** | 75.16 (10.50) | 74.64 (9.67) | 0.425 | 75.67 (10.13) | 75.93 (10.51) | 0.597 |
| **SBP, mean (SD), mmHg** | 142.88 (18.58) | 142.80 (19.46) | 0.953 | 145.54 (17.48) | 146.22 (19.06) | 0.432 |
| **DBP, mean (SD), mmHg** | 83.87 (12.13) | 84.21 (11.97) | 0.655 | 86.01 (12.06) | 86.90 (12.49) | 0.126 |
| **mRS ≤2 at randomization, n(%)** | 275 (56.4) | 278 (57.4) | 0.782 | 484 (53.6) | 520 (57.5) | 0.103 |
| **NIHSS at randomization, mean (SD)** | 5.78 (2.30) | 6.02 (2.48) | 0.109 | 5.95 (2.39) | 5.85 (2.29) | 0.371 |
| **IS, n (%)** | 75 (15.4) | 81 (16.7) | 0.622 | 153 (16.9) | 173 (19.1) | 0.250 |
| **TIA, n (%)** | 1 (0.2) | 6 (1.2) | 0.126 | 4 (0.4) | 6 (0.7) | 0.752 |
| **VSA, n (%)** | 3 (0.6) | 15 (3.1) | 0.008^**^ | 22 (2.4) | 20 (2.2) | 0.873 |
| **Hyperlipidemia, n (%)** | 18 (3.7) | 22 (4.5) | 0.609 | 48 (5.3) | 59 (6.5) | 0.322 |
| **Hypertension, n (%)** | 250 (51.2) | 253 (52.3) | 0.794 | 544 (60.2) | 536 (59.3) | 0.715 |
| **Diabetes, n (%)** | 115 (23.6) | 93 (19.2) | 0.115 | 233 (25.8) | 256 (28.3) | 0.250 |
| **Current smoking, n (%)** | 132 (27.0) | 146 (30.2) | 0.315 | 218 (24.1) | 239 (26.4) | 0.285 |
| **Current drinking, n (%)** | 63 (12.9) | 68 (14.0) | 0.670 | 132 (14.6) | 115 (12.7) | 0.269 |
| **pre-mRS =0, n (%)** | 342 (70.1) | 349 (72.1) | 0.532 | 659 (73.0) | 661 (73.1) | 0.989 |

**Abbreviations:** BMI, body mass index; DBP, diastolic blood pressure; IS, ischaemic stroke; mRS, modified Rankin scale; NIHSS, National Institutes of Health Stroke Scale; PNS, *Panax Notoginseng* Saponins; SBP, systolic blood pressure; TIA, transient ischemic attack; VSA, vasospastic angina; WC, waist circumference.

**P* value＜0.05；***P* value＜0.01; ****P* value＜0.001.
