# Supplementary material for: Efficacy of Panax notoginseng saponins on functional outcome in obese patients with acute ischemic stroke
Source: J Ginseng Res. 2026 Feb 6;50(3):100991. doi: 10.1016/j.jgr.2026.100991 (PMC13149892; doi:10.1016/j.jgr.2026.100991)
Supplement: Multimedia component 6 [file mmc6.docx]

**Table S3 Baseline Characteristics Stratified by BMI and WC**

|  | **Lower BMI + Normal WC** | | | **Higher BMI + Normal WC** | | | **Lower BMI + Abdominal Obesity** | | | **Higher BMI + Abdominal Obesity** | | |
| --- | --- | --- | --- | --- | --- | --- | --- | --- | --- | --- | --- | --- |
|  | **(n=639)** | | | **(n=333)** | | | **(n=520)** | | | **(n=1287)** | | |
|  | **PNS** | **Placebo** | ***P* value** | **PNS** | **Placebo** | ***P* value** | **PNS** | **Placebo** | ***P* value** | **PNS** | **Placebo** | ***P* value** |
|  | **(n=320)** | **(n=319)** |  | **(n=168)** | **(n=165)** |  | **(n=265)** | **(n=255)** |  | **(n=638)** | **(n=649)** |  |
| **Male Sex, n (%)** | 234(73.1) | 236(74.0) | 0.876 | 116(69.0) | 124(75.2) | 0.263 | 154(58.1) | 153(60.0) | 0.728 | 387(60.7) | 448(69.0) | 0.002^**^ |
| **Age, mean (SD), years** | 62.66(8.76) | 62.55(9.66) | 0.876 | 61.50(9.12) | 59.50(9.34) | 0.049^*^ | 60.66(8.52) | 62.07(8.28) | 0.057 | 59.85(9.65) | 59.41(9.48) | 0.408 |
| **Heart rate, mean (SD)** | 75.46(10.58) | 74.72(10.08) | 0.365 | 74.59(10.36) | 74.50(8.85) | 0.930 | 76.91(11.17) | 76.25(10.41) | 0.487 | 75.16(9.63) | 75.81(10.55) | 0.253 |
| **SBP, mean (SD), mmHg** | 142.27(19.43) | 141.97(20.01) | 0.849 | 144.04(16.84) | 144.42(18.31) | 0.843 | 143.38(17.92) | 146.20(18.41) | 0.077 | 146.44(17.23) | 146.22(19.32) | 0.832 |
| **DBP, mean (SD), mmHg** | 83.61(12.23) | 82.83(11.71) | 0.410 | 84.36(11.96) | 86.90(12.03) | 0.055 | 84.35(11.97) | 85.25(12.71) | 0.406 | 86.70(12.04) | 87.54(12.35) | 0.216 |
| **mRS ≤2 at randomization, n(%)** | 180(56.2) | 177(55.5) | 0.909 | 95(56.5) | 101(61.2) | 0.451 | 144(54.3) | 145(56.9) | 0.624 | 340(53.3) | 375(57.8) | 0.118 |
| **NIHSS at randomization, mean (SD)** | 5.97(2.46) | 5.99(2.42) | 0.923 | 5.40(1.91) | 6.08(2.61) | 0.007^**^ | 6.11(2.49) | 5.97(2.37) | 0.521 | 5.89(2.34) | 5.81(2.25) | 0.534 |
| **IS, n (%)** | 46(14.4) | 56(17.6) | 0.323 | 29(17.3) | 25(15.2) | 0.709 | 36(13.6) | 53(20.8) | 0.039^*^ | 117(18.3) | 120(18.5) | 1.000 |
| **TIA, n (%)** | 1(0.3) | 4(1.3) | 0.367 | 0(0.0) | 2(1.2) | 0.470 | 2(0.8) | 2(0.8) | 1.000 | 2(0.3) | 4(0.6) | 0.698 |
| **VSA, n (%)** | 3(0.9) | 13(4.1) | 0.022^*^ | 0(0.0) | 2(1.2) | 0.470 | 8(3.0) | 5(2.0) | 0.623 | 14(2.2) | 15(2.3) | 1.000 |
| **Hyperlipidemia, n (%)** | 11(3.4) | 20(6.3) | 0.138 | 7(4.2) | 2(1.2) | 0.185 | 5(1.9) | 16(6.3) | 0.020^*^ | 43(6.7) | 43(6.6) | 1.000 |
| **Hypertension, n (%)** | 152(47.5) | 149(46.7) | 0.904 | 98(58.3) | 104(63.0) | 0.444 | 133(50.2) | 130(51.0) | 0.926 | 411(64.4) | 406(62.6) | 0.525 |
| **Diabetes, n (%)** | 70(21.9) | 60(18.8) | 0.387 | 45(26.8) | 33(20.0) | 0.183 | 58(21.9) | 73(28.6) | 0.095 | 175(27.4) | 183(28.2) | 0.806 |
| **Current smoking, n (%)** | 89(27.8) | 99(31.0) | 0.420 | 43(25.6) | 47(28.5) | 0.638 | 69(26.0) | 64(25.1) | 0.885 | 149(23.4) | 175(27.0) | 0.153 |
| **Current drinking, n (%)** | 40(12.5) | 45(14.1) | 0.630 | 23(13.7) | 23(13.9) | 1.000 | 38(14.3) | 36(14.1) | 1.000 | 94(14.7) | 79(12.2) | 0.206 |
| **pre-mRS =0, n (%)** | 219(68.4) | 232(72.7) | 0.270 | 123(73.2) | 117(70.9) | 0.729 | 181(68.3) | 175(68.6) | 1.000 | 478(74.9) | 486(74.9) | 1.000 |

**Abbreviations:** BMI, body mass index; DBP, diastolic blood pressure; IS, ischaemic stroke; mRS, modified Rankin scale; NIHSS, National Institutes of Health Stroke Scale; PNS, *Panax Notoginseng* Saponins; SBP, systolic blood pressure; TIA, transient ischemic attack; VSA, vasospastic angina; WC, waist circumference.

**P* value＜0.05；***P* value＜0.01; ****P* value＜0.001.
